# A Comprehensive Review of Medicarpin: A Phytoalexin with Therapeutic Potential

**DOI:** 10.1021/acsomega.5c08170

**Published:** 2025-11-07

**Authors:** Matheus Hikaru Tanimoto, Aline Mayrink de Miranda, Jennyfer Andrea Aldana-Mejía, Luciana Silva de Araújo, Ana Maria de Freitas Pinheiro, Ana Fernanda Guimarães Trindade, Júlia Mina Fernandes, Samir A. Ross, Jairo Kenupp Bastos

**Affiliations:** † School of Pharmaceutical Sciences of Ribeirão Preto, University of São Paulo, Ribeirão Preto, SP 14040-903, Brazil; ‡ Faculty of Pharmaceutical Sciences, University of São Paulo, São Paulo, SP 05508-000, Brazil; § National Center for Natural Products Research, School of Pharmacy, The University of Mississippi, Oxford, Mississippi 38677, United States; ∥ Department of Biomolecular Sciences, Division of Pharmacognosy, School of Pharmacy, University of Mississippi, Oxford, Mississippi 38677, United States

## Abstract

Initially, research on medicarpin focused on its role in crop protection, including gene modifications, allelopathic effects, antifungal potential, and biosynthetic mechanisms. Later, various biological activities were demonstrated through *in vitro* and *in vivo* studies, including anti-inflammatory, antimicrobial, antioxidant, antiproliferative, and neuroprotective properties, with notable effects on bone metabolism. Recent progress has been made in developing synthetic routes for obtaining medicarpin and in analytical methods for its analysis and quantification. Studies on preclinical pharmacokinetics and advances in alternative synthesis of medicarpin are at the forefront of current research. Advancements in stability, toxicity, pharmaceutical technologies in formulations, and potential clinical applications are necessary but remain challenging. This review aims to provide critical insights and compile the main botanical, chemical, and therapeutic aspects of medicarpin, as well as the challenges and perspectives for the biomedical and pharmaceutical fields.

## Graphical abstract

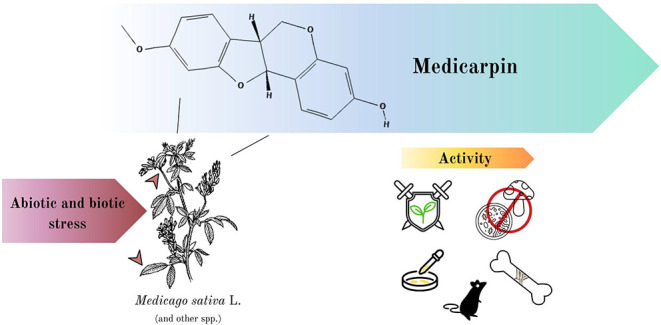

## 1 Introduction

Unlike other living organisms, plants are sessile, meaning they cannot escape threats such as herbivory or adverse environmental conditions.^1^ Consequently, they have evolved sophisticated chemical defense mechanisms to ensure survival.^2^ Over time, these biochemical defense pathways have been selectively propagated, resulting in highly specialized chemical responses that vary among different plant families. Among these responses, phytoalexins represent a critical class of defense compounds synthesized in response to biotic and abiotic stressors. Furthermore, the diversity in these chemical arsenals across plant families suggests evolutionary specificity shaped by distinct ecological pressures.^3–5^

One of the primary classes of secondary metabolites classified as phytoalexins is isoflavonoids. Within this group, the pterocarpan medicarpin has garnered significant attention since the 1970s, especially in phytopathology, due to its important role in plant chemical defense, particularly in the Fabaceae family. Medicarpin is a pterocarpan phytoalexin predominantly produced by species within the Fabaceae family.^3,6,7^

Initially, extraction and isolation methods for medicarpin focused on plant roots, where studies demonstrated its preferential accumulation in tissues such as *Medicago sativa* L. Research has shown that plants stabilize the medicarpin molecule and increase its water solubility through glycosylation, facilitating its rapid transport and storage in vacuoles as a potentially toxic defense compound.^8,9^ Nonetheless, under certain stress conditions, such as copper exposure, plants may synthesize free medicarpin de novo, despite having glycosylated reserves.^8–11^

Decades later, beyond its ecological role, studies began to explore medicarpin’s therapeutic potential. Medicarpin has shown promising activity across various medicinal applications, including its antimicrobial,^6,12–15^ anti-inflammatory,^16^ antioxidant,^17^ antiproliferative, and pro-apoptotic effects in cancer cells,^18–22^ as well as neuroprotective Properties.^23–27^ These diverse bioactivities make medicarpin an appealing candidate for in-depth biomedical and pharmaceutical research, given its versatility and potential as a natural bioactive agent. Among its bioactivities, one of the most extensively studied is its effect on bone metabolism, with both in vitro and in vivo studies demonstrating significant implications.^28–34^ Advanced preclinical pharmacokinetic studies, including assessments of absorption, tissue distribution, excretion, and bioavailability, alongside validated bioanalytical methods, provide a solid foundation for future research.^35–37^

Despite advances in understanding its bioactivity, mechanisms of action, agricultural importance, allelopathic effects, influence on transporters, preclinical studies, advanced analytical methods, and total synthesis, several critical knowledge gaps and technological challenges remain unaddressed. Issues such as toxicity, unexplored *in vitro* activities, and studies on physicochemical stability are essential for advancing to clinical safety and efficacy trials, as well as for technological and pharmacological innovations that improve both its administration and, consequently, the bioavailability of medicarpin. As a phytoalexin produced by plants in response to environmental challenges, widely present in dietary legumes, essential to agriculture, and with notable therapeutic and medicinal potential, this review aims to compile the main findings on medicarpin, a promising phytoalexin.

As part of the research strategy and selection criteria for this work, publications were selected through searches in primary online databases (ScienceDirect, Web of Science, PubMed, and Google Scholar). The keyword “medicarpin” was used, restricted to the title section of the search engines. Duplicate articles were excluded, and only research articles published in English were included. No time filter was applied. Under these conditions, articles discussing medicarpin’s botanical origin, chemical characterization, and biological activities were ultimately considered. This article aims to help readers understand the role and significance of research on medicarpin, while also providing a foundation for future studies that can further explore and harness the full therapeutic potential of this phytoalexin.

## 2 Botanical Origins and Sources

Medicarpin is a pterocarpan derived from isoflavonoids, primarily associated with leguminous plants. The Fabaceae family (also known as Leguminosae) is the third largest plant family and the second most economically important, with nearly 20,000 known species, including *Glycine max* (L.) Merr. (soybean) and *Arachis hypogaea* L. (peanut).^38^ Isoflavonoids are predominantly found in members of the Fabaceae family,^39^ leading to the assumption that both pterocarpan and medicarpin are also associated with this family. A review of 46 papers on botanical sources of medicarpin identified a total of 19 species across 14 different genera, all belonging to the subfamily Faboideae Rudd,^40^ which is the largest subfamily within Fabaceae. This suggests that medicarpin may be a characteristic compound of the subfamily rather than the entire family. Of the 19 species, *Medicago sativa* L. was the most commonly used, followed by *Trifolium repens* L. and *Trigonella foenum-graecum* L. *M*. *sativa* L., also known as lucerne or alfalfa, is a perennial crop primarily cultivated for agricultural purposes, similar to *T*. *foenum-graecum* L. and *T*. *repens* L. (Table 1).

**Table 1:** Botanical Origins, Species, and Source Tissues of Medicarpin

family: fabaceae			
subfamily: fabanoideae			
species	tissue source	medicarpin extracted	references
*Arachis hypogaea* L.	Infected tissue and leaves	–	^41^
	Infected Leaflet	15 μg–5.5 mg/g fresh wt (after 20 weeks)	^42^
*Canavalia ensiformis* (L.) DC	Infected Cotyledons		^43^
	–		^44^
	Infected Cotyledon	–	^45^
*Canavalia lineata* (Thunb.) DC	Pods	22.5 mg (from 1.2 kg)	^25^
*Cicer arietinum* L.	Seed	–	^46^
	Suspension cell culture	–	^47^
	–		^48^
*Dalbergia congestiflora* Pittier	Wood (heartwood)	–	^14^
*Dalbergia ecastaphyllum* (L.) Taub.	Resin and Stems	12% g/100 g (Resin) 2.89% g/100 g (Stems)	^49^
*Dalbergia odorifera* T. Chen	Wood (heartwood)	–	^16^
*Glycyrrhiza glabra* L.	Root	–	^50^
*Glycyrrhiza uralensis* Fisch.	Root	–	^50^
*Hedysarum polybotrys* Hand.-Mazz.	Root	297.5 mg (from 4.8 kg)	^23^
*Maackia amurensis* Rupr	Radicle	–	^37^
*Medicago sativa* L.	Suspension cell culture	–	^51^
	Leaflet	–	^52^
	Root	250 μg/g fresh wt (after 48 h)	^53^
	Sprouts (newly germinated)	1.64–9.61 μg/g fresh wt (after 48 h)	^54^
	Rhizome	5.3 mg (from 100 mg of medicarpin-β-d-glucoside)	^55^
	Whole plant	–	^56^
	Leaflet, root, and nodules	–	^57^
	Callus (from leaflet)	–	^58^
	Leaves	–	^64^
	Leaves	–	^59^
	Leaflet	–	^7^
	Seedling	–	^43^
	Suspension cell culture	–	^60^
	Suspension cell culture	–	^12^
	Callus and suspension cell culture	–	^53^
	Leaves	1–2 μg/g fresh wt	^6^
	Callus (from seeds)	–	^61^
*Medicago truncatula* Gaertn.	Radicle	–	^62^
	Leaves	–	^13^
*Melilotus officinali*s (L). Lam.	Leaflets	–	^45^
*Robinia pseudoacacia* L.	Root	–	^17^
*Sophora japonica* L.	Leaves	2.4 μg/mL (after 72 h)	^63^
	Infected tissue and leaves	–	^41^
*Trifolium repens* L.	Leaflet	1–2 μg/g fresh wt	^64^
	Callus (from hypocotyl tissue)	8 μg (from 240 mL inoculated solution)	^65^
	Leaflet	–	^66^
	Leaves	–	^6^
*Trigonella foenum-graecum* L.	Suspension cell culture	–	^11^
	Seedlings		^9^
	Seedling root	–	^10^
	Seedling	–	^67^
*Vicia jaba* L.	Endocarp (pod endocarp)	19.5 μg/g fresh tissue (after 96 h)	^68^
*Trifolium pratense* L.^a^	–	–	^69^
*Medicago sativa* L.^a^	–	–	^6^
Jamaican multifloral propolis	–	2.4 μg/mL (after 72 h)	^15^

a – paper did not specify if medicarpin was extracted.

As mentioned throughout the text, medicarpin has been identified in various tissues and parts of plants, including cotyledons, seedlings, heartwood, and the endocarp of pods. However, this compound is not only found in plants but also in propolis. As described by Ccana-Ccapatinta and collaborators^70^ medicarpin, like other compounds, can be incorporated into propolis through the feeding habits of bees, highlighting the importance of botanical sources in such evaluations. In a study by Daugsch and collaborators,^71^ *Dalbergia ecastaphyllum* (L.) Taub. (Fabaceae) was identified as the primary source of the chemical composition in red propolis, with medicarpin being isolated as a major compound.

The literature reports *D*. *ecastaphyllum* as a source of medicarpin. Besides red propolis, other types of propolis containing medicarpin have been investigated, and their botanical sources have been thoroughly examined. Jamaican propolis has been cited as a source in one study, although its botanical origin was not provided. Current data suggests that species from the Faboideae subfamily may be the source for the bees responsible for producing this propolis.

## 3 Phytoalexins and Their Role in Legume Defense

### 3.1 Phytoalexins: General Characteristics

Phytoalexins are antimicrobial compounds synthesized by plants in response to pathogenic invasion or environmental stress, acting as a primary line of biochemical defense.^3^ Among these, medicarpin, a pterocarpan phytoalexin, is prominent in legumes such as alfalfa (*Medicago sativa*) and red clover (*Trifolium pratense*).^6,12^ Induced by exposure to biotic stressors like fungi and abiotic stressors such as heavy metals, medicarpin disrupts pathogen development by accumulating in infected plant tissues and preventing the spread of pathogens.^10,13^

Understanding the induction mechanisms and functional roles of medicarpin is crucial for improving plant resilience, especially in economically significant crops prone to pathogen stress, similar to other phytoalexins.^5,68,72–74^ Harnessing the natural properties of medicarpin offers a pathway to developing sustainable strategies for enhancing plant defense while minimizing dependence on synthetic chemicals. Studying medicarpin in leguminous plants also opens avenues for selective breeding and bioengineering to optimize crop resilience and productivity.^12,50^

### 3.2 Medicarpin: A Key Player in Pathogen Resistance

In the early 1980s, studies began to identify medicarpin as a phytoalexin playing a vital role in plant immunity, directly inhibiting pathogen growth and engaging signaling pathways to strengthen the plant’s defense response. This pterocarpan has been identified with this role in several Fabaceae plants, including *Medicago sativa* (alfalfa),^54,60,75^ *Cicer arietinum* (chickpea),^46^ *Pisum sativum* (pea),^76^ *Arachis hypogaea* (groundnut),^42^ *Trifolium repens* (white clover),^64^ *Sophora japonica* (Chinese scholar tree),^63^ among others.

Medicarpin’s role in plant defense was underscored by its increased biosynthesis in response to pathogenic fungal infections.^42,54,63^ For example, alfalfa plants activate medicarpin production in response to fungal pathogens such as *Phytophthora megasperma* and *Verticillium albo-atrum*, where this compound accumulates at infection sites, disrupting fungal colonization.^12,43,52,58^

Research on *Medicago truncatula* indicates that medicarpin not only accumulates around fungal infection sites but also interacts with the salicylic acid (SA) pathway, which plays a crucial role in mounting an effective immune response against pathogens such as powdery mildew (*Erysiphe pisi*). Recent studies have begun to explore the medicarpin biosynthetic response in *M*. *truncatula* genotypes with varying resistance to *E*. *pisi*. In resistant genotypes, gene expression occurs earlier (6–12 h postinfection) compared to susceptible genotypes (12–24 h postinfection), suggesting a correlation with the timing of pathogen penetration attempts.^13^

However, medicarpin’s efficacy varies among pathogens, as some species have evolved detoxification mechanisms to degrade it.^67,75,77^ Species of the *Fusarium* genus, for example, can demethylate and hydroxylate medicarpin, thereby reducing its toxicity and circumventing its antifungal activity.^75,78–80^ This pathogen adaptation underlines a critical limitation: while medicarpin is effective against certain pathogens, its utility may diminish as pathogens develop resistance mechanisms, thus impacting overall crop resilience.

### 3.3 Medicarpin’s Differential Response to Biotic and Abiotic Stress

As mentioned, medicarpin can be produced and accumulated in response to stress in plants, regulated by the binding of fungal or bacterial metabolites, as well as other biotic elicitors, to plant cell receptors.^65^ Interestingly, these interactions can activate three possible defense mechanisms. One of these is the direct activation of isoflavonoid biosynthesis. However, some studies have revealed that once plant cell receptors detect the trigger, the accumulation of defense phytohormones, such as salicylic acid (SA) and jasmonic acid (JA), can also be activated in certain plant genotypes.^13^ Furthermore, medicarpin’s accumulation at the infection sites on the plasma membrane of plant cells increases SA levels, and this interaction may trigger H_2_O_2_ production, thereby reducing fungal penetration and colony formation (Figure 1).

**Figure 1:**
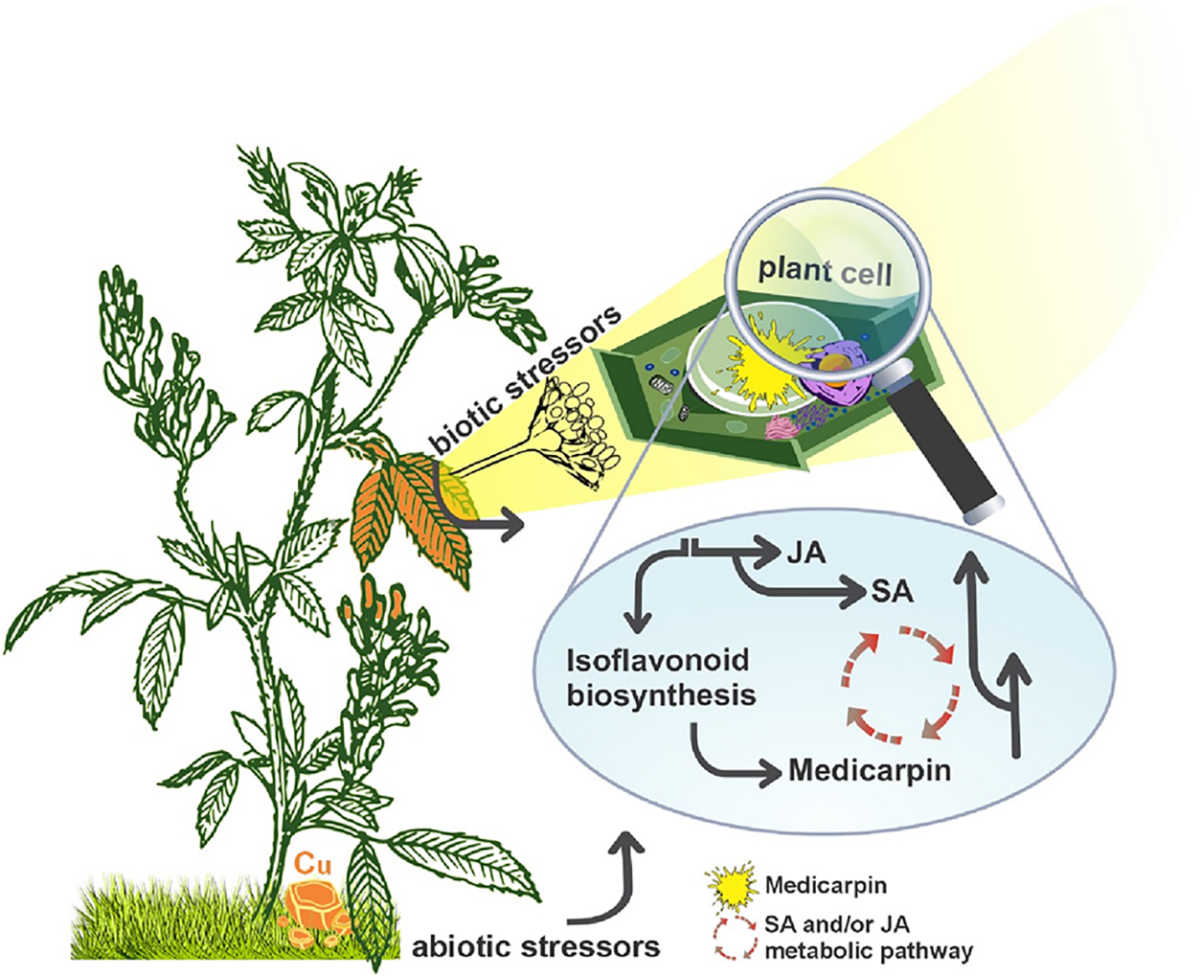
Potential Mechanisms of Phytoalexin-Mediated Legume Defense: Metabolic Pathways Model. **JA:** jasmonic acid. **SA:** salicylic acid. Image adapted from [www.pixabay.com], with modifications (drawn in CorelDRAW).

Another interesting abiotic stress recently described is the discovery of the insect *Agrilus propolis* Migliore, Curletti, and Casari sp. nov. This insect completes its maturation inside *Dalbergia ecastaphyllum*, emerging through a hole it creates in the plant’s stem. Although this insect-plant interaction requires further investigation to fully understand this intriguing relationship, it is known that, as a result of the wound, the plant exudes a characteristic red resin. This resin becomes accessible to *Apis mellifera* L. bees, thereby enabling them to produce Brazilian red propolis^81^

To date, studies on abiotic stress have focused on exposing medicarpin-producing plants to metals. For example, exposure to copper can initiate medicarpin synthesis in fenugreek (*Trigonella foenum-graecum*).^10,11^ In *Arachis hypogaea* (groundnut), medicarpin levels rise upon treatment with copper­(II) sulfate.^42^ Lead (Pb) exposure in *Medicago sativa* decreases medicarpin concentration in roots while promoting exudation, with differential effects on the expression of genes and enzymes involved in flavonoid biosynthesis and plant defense responses, such as phenylalanine ammonia-lyase and viral resistance.^56^

Under these abiotic stresses, medicarpin aids in detoxifying harmful metals and mitigating cellular damage. Medicarpin appears to function by binding to these metals or by being excreted from root cells, thereby limiting metal accumulation in plant tissues and reducing oxidative stress.^10,11^ To date, it remains unclear whether abiotic stressors also trigger the activation of other defense compounds. However, it is plausible that, in this context, the plant prioritizes the detoxification of the stressors.

### 3.4 Medicarpin as an Allelopathic Agent

Beyond its antimicrobial role, medicarpin exhibits allelopathic effects, particularly in mature alfalfa plants where it inhibits the germination and growth of nearby seedlings.^59^ This phenomenon likely provides a competitive advantage by reducing competition for resources such as water and nutrients from other plants. In alfalfa seedlings, medicarpin functions as a potent allelopathic agent, significantly inhibiting seedling growth at concentrations as low as 5 × 10^–8^ mol/seed. This autotoxic effect, where mature plants suppress the germination and growth of nearby seedlings, confers a competitive advantage by reducing resource competition. Exogenous application of medicarpin in agar bioassays resulted in a 39% reduction in seedling length after 72 h, with the compound being absorbed and metabolized by the seedlings within 44 h, after which growth resumed at normal rates.^59^ The allelopathic behavior of medicarpin raises ecological and agricultural questions, particularly regarding its impact on soil composition and neighboring plant species. Long-term planting of medicarpin-producing crops could alter soil biochemistry, potentially affecting crop rotation and intercropping practices, as well as biodiversity in agricultural systems.

### 3.5 Potential Applications and Challenges in Agriculture

Medicarpin presents a promising natural alternative to pesticides within the Fabaceae family, suggesting that it may reduce the need for chemical applications by enhancing the plants’ intrinsic resistance. However, the literature still lacks sufficient studies to confirm this potential. Advances in bioengineering and selective breeding to develop medicarpin-rich crops could significantly benefit sustainable agriculture by improving yield resilience without external inputs.

Other methods for medicarpin production have been explored. Transcriptome analyses have identified 176 genes in *Glycyrrhiza uralensis* and *Glycyrrhiza glabra* involved in medicarpin biosynthesis. The increasing interest in synthetic biology has led to advances in engineering microbial systems, such as *Saccharomyces cerevisiae*, to produce medicarpin more efficiently. When the eight most highly expressed genes were introduced into *S*. *cerevisiae* strain DW11, medicarpin production was enhanced, demonstrating how omics technology and synthetic biology can advance our understanding of and capacity for phytoalexin biosynthesis.^50^

Nevertheless, several challenges remain in practical applications. Pathogen resistance, environmental degradation of medicarpin, and the compound’s allelopathic effects could complicate its agricultural deployment. Additionally, medicarpin’s stability and efficacy may be variable under field conditions, influenced by factors such as soil composition, temperature, and pathogen diversity. Integrating phytoalexins into crop management systems, therefore, requires a nuanced understanding of their interactions with environmental and biological factors. Some studies have focused on how phytopathogenic microorganisms can degrade medicarpin into less toxic molecules or completely degrade its chemical structure.^78–80^ Hence, there is a significant gap in understanding the stability of medicarpin under environmental or abiotic factors.

This review demonstrates medicarpin’s critical role in legume defense through its antimicrobial, allelopathic, and detoxification properties. While promising as a natural defense molecule, medicarpin’s effectiveness is moderated by pathogen adaptability, environmental factors, and its impact on surrounding plant communities.

Future research could focus on genetic modifications that enhance medicarpin’s resilience against detoxifying pathogens or enable higher production under variable conditions. Exploring sustainable agricultural practices that capitalize on medicarpin’s benefits while addressing ecological impacts, such as its allelopathic effects, will be essential for its successful integration into crop protection strategies. Moreover, advancements in synthetic biology could support scalable medicarpin production for both agricultural and medicinal applications, paving the way for its expanded role in plant-based resilience solutions.

## 4 Exploring the Chemical Nature of Medicarpin

### 4.1 Physical and Chemical Properties

Medicarpin is a naturally occurring compound classified as a pterocarpan, characterized by its fused furan ring structure, which forms through the cyclization between the C-4 carbonyl group and the C-2′ positions of a 2′-hydroxyisoflavanone.^82^ Due to the presence of asymmetric carbons at positions 6a and 11a, four possible diastereomeric structures can exist. However, the 6a–11a bond was confirmed as a cis-fusion of the two heterocyclic rings, limiting the naturally occurring forms to a single pair of enantiomers^63^ (Figure 2).

**Figure 2:**
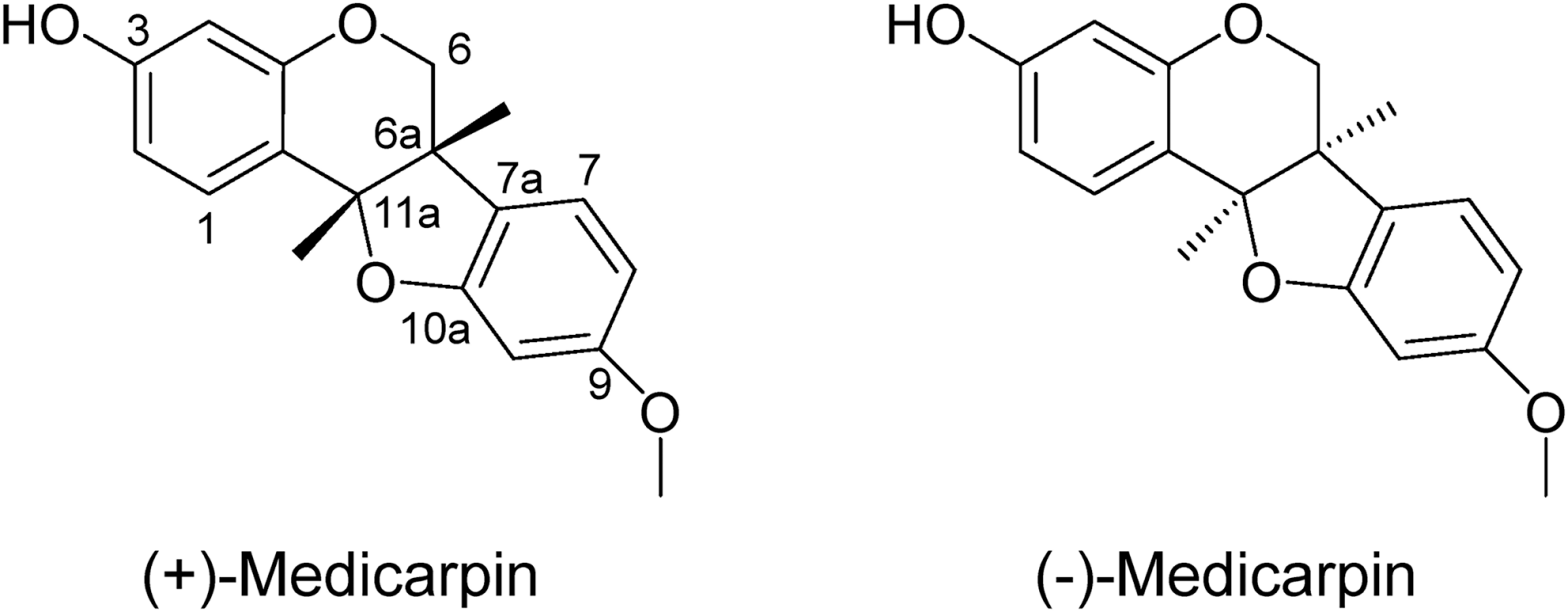
Structures of (+) and (−) medicarpin enantiomers.

Its physical and chemical characteristics have been extensively studied since the 1970s using various analytical techniques. Sakagami, Kumai, and Suzuki^55^ utilized Nuclear Magnetic Resonance (NMR), Infrared Spectroscopy (IR), Mass Spectrometry (MS), Ultraviolet Spectroscopy (UV), and Optical Rotatory Dispersion (ORD) methods. Key MS data include a molecular ion at *m*/*z* 270, with fragment ions at 255, 197, 161, 148, 147, 137, and 135, confirming its molecular weight and fragmentation pattern. UV spectra in methanol show absorption maxima at 282 nm (ε = 4977) and 287 nm (ε = 5526), indicative of conjugated systems typical of pterocarpans. The IR spectrum presents significant absorption bands at 3390 cm^–1^ (hydroxyl group) and 1619, 1588, 1493 cm^–1^, which correspond to aromatic ring vibrations.

The ^1^H NMR data in deuterated chloroform reveal the presence of methoxy and aromatic protons, with chemical shifts at 3.49–3.75 ppm for protons H6α and H6ax, 4.17–4.29 ppm for 6-Heq, 5.48 ppm for H11a, and aromatic protons appearing between 6.39 and 7.37 ppm, suggesting a highly conjugated aromatic system. Similar data was obtained in deuterated acetone (42). Furthermore, ORD analysis shows notable optical activity with a specific rotation [α]­28 value of −120° at 590 nm, indicating the compound’s chirality (55).

Hargreaves et al.^68^ further confirmed medicarpin’s structure in *Vicia faba* L., reporting a melting point of 129–130 °C and optical activity ([α]*D*20 = −214°). They also provided UV maxima in ethanol at 287 nm (log ε = 3.94), 282 nm (3.88), 226 nm (4.14), and 210 nm (4.53), corroborating earlier findings. Similarly, Strange et al.^42^ identified medicarpin from infected *Arachis hypogaea* leaflets using MS, yielding a molecular ion at *m*/*z* 270, and ^1^H NMR data showing characteristic aromatic and methoxy proton shifts.

Sharma et al.^31^ synthesized and identified medicarpin, reporting a higher melting point (188–190 °C) and providing detailed NMR (both ^1^H and ^13^C) and MS exact mass calculated for C16H14O4 [M + H]+, 271.0970, found 271.0965. Williams et al.^15^ differentiated between the enantiomers of medicarpin using high-resolution ESI TOF-MS and NMR, showing that the only structural difference between (+)-medicarpin and (−)-medicarpin lies in the positions of 6a and 11a, which impacts their optical rotation and biological properties. According to the NMR analysis, (+)-medicarpin is characterized by ^1^H signals at 3.56 ppm (m, H-6β), 4.22 ppm (dd, 10.8, 4.8, H-6α), 3.51 ppm (m, H-6a), and 5.50 ppm (d, 6.7, H-11a) and 13C NMR signals at 66.5 for C6, 40.0 for C6a, and 78.9 for C11a.

Overall, medicarpin’s well-documented physical and chemical characteristics include its molecular weight of 270 g/mol, specific UV absorption, NMR spectral data, and distinctive optical rotation. The challenges in distinguishing between its isomers and enantiomers underline the importance of advanced analytical techniques, such as high-resolution mass spectrometry and detailed NMR analysis, to ensure accurate identification and structural elucidation of this biologically active compound.

### 4.2 Biosynthetic Pathway

Medicarpin is a product of the well-characterized phenylpropanoid pathway, which synthesizes various plant monolignols, stilbenes, coumarins, and flavonoids. Medicarpin biosynthesis begins with the amino acid l-phenylalanine and proceeds through the phenylpropanoid pathway, ultimately branching into the isoflavonoid pathway. Key enzymes, including cytochrome P450 variants, catalyze the steps leading to the production of medicarpin.^50,60^

In 1981, the isoflavones formononetin, 2′-hydroxyformononetin, and vestitone were identified as biosynthetic precursors of medicarpin in *Trifolium repens*.^64^ Elicitors such as *p*-chloromercuribenzoic acid (PCMBA) and HgCl_2_ were shown to stimulate medicarpin biosynthesis in *T*. *repens*.^65^ Additionally, when *Medicago sativa* was exposed to fungal elicitors from *Colletotrichum lindemuthianum*, both the cells and culture medium exhibited elevated levels of medicarpin, along with the induction of specific biosynthetic enzymes, particularly cytochrome P450 enzymes.^60^ Enzymes such as phenylalanine ammonia-lyase, cinnamic acid 4-hydroxylase, chalcone isomerase, and chalcone synthase were produced in increased amounts during phytoalexin accumulation when elicitors induced pterocarpan production.^46,65^

In *Medicago truncatula*, the biosynthesis of medicarpin involves the sequential formation of phenylpropanoid, flavonoid, and isoflavonoid cores before final pterocarpan production.^62^ The pathway begins with the conversion of l-phenylalanine to 4-coumaroyl-CoA, catalyzed by phenylalanine ammonia-lyase (PAL), cinnamate-4-hydroxylase, and 4-coumarate-CoA ligase (Figure 3). The reaction between 4-coumaroyl-CoA and malonyl-CoA, mediated by chalcone synthase and chalcone reductase, produces isoliquiritigenin, which is a precursor for 5-deoxyisoflavonoids synthesized by isoflavone synthase. Formononetin is then produced, and through isoflavone 2′-hydroxylase (I2′H) activity, 2′–OH formononetin is formed.

**Figure 3:**
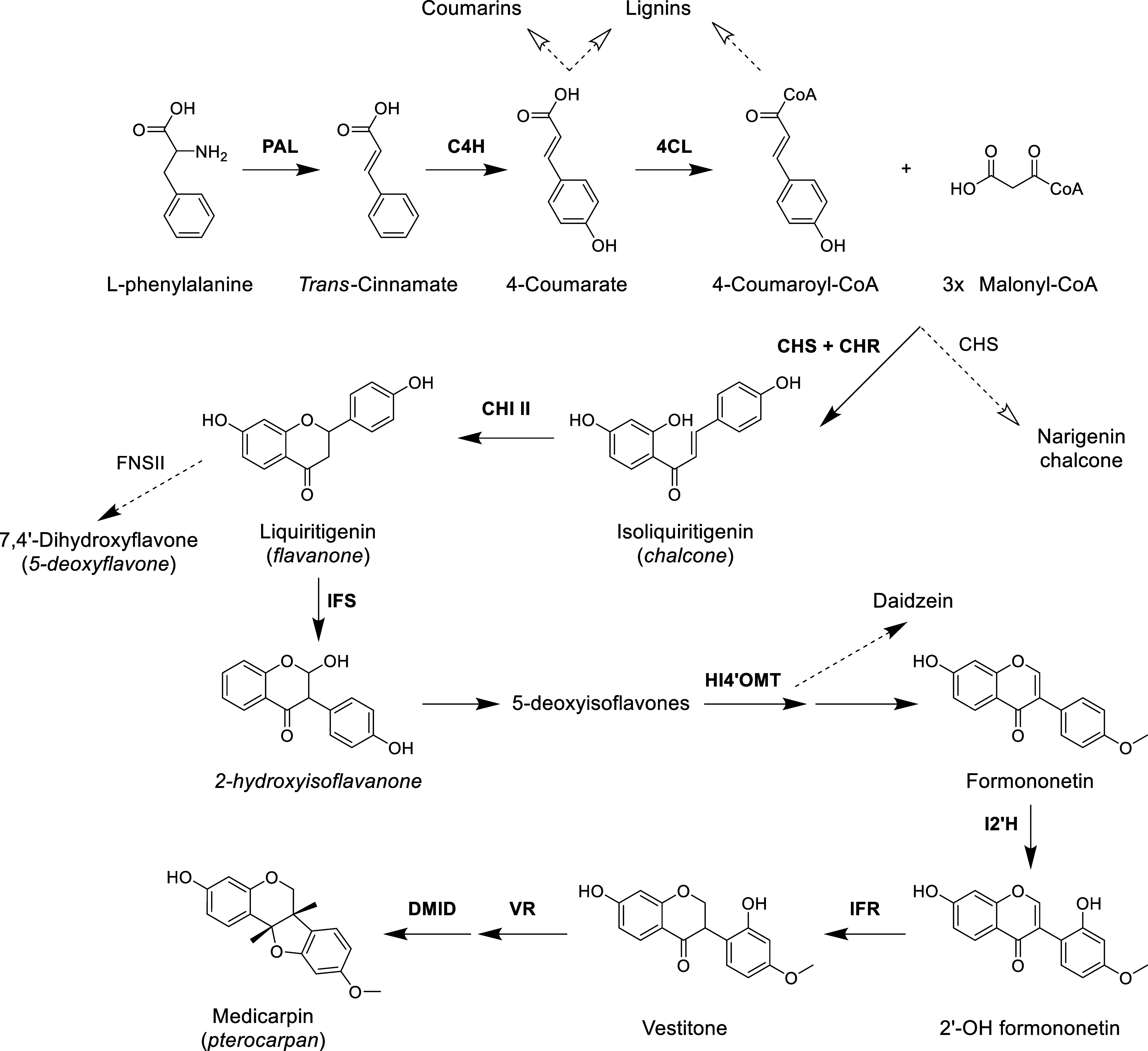
Scheme of medicarpin biosynthetic pathway in *M*. *truncatula*. PAL, phenylalanine ammonia-lyase; C4H, cinnamic acid 4-hydroxylase; 4CL, 4-coumarate-CoA ligase; CHS, chalcone synthase; CHR, chalcone reductase; CHI II, chalcone isomerase II; IFS, isoflavone synthase; HI4′OMT, isoflavone 4′-O-methyltransferase; I2′H, isoflavone 2′-hydroxylase; IFR, isoflavone reductase; VR, vestitone reductase; DMID, 4′-methoxyisoflavanol dehydratase; FNSII, flavanone synthase II. Adapted from Biała et al.^62^

Radiolabeling studies have shown that medicarpin is derived from formononetin via specific E-addition of hydrogen to formononetin’s double bond, catalyzed by isoflavone reductase, which reduces 2′-hydroxy-formononetin to (3R)-vestitone, a 2′-hydroxylated isoflavanone (76). An unusual case in *Sophora japonica* inoculated with *Helminthosporium carbonum* produced both (+) and (−) enantiomers, with (+) being predominant. This suggests that either E and Z reductions occur or that a single reduction enzyme acts bidirectionally on the isoflavone molecule.^63^

In *Medicago sativa*, the final step in medicarpin biosynthesis involves two enzymes: vestitone reductase, which reduces vestitone to 7,2′-dihydroxy-4′-methoxy-isoflavanol in a NADPH-dependent reaction (12,53), and DMI dehydratase, which facilitates water loss and ring closure to yield the stereoisomer (−) or (6aR,11aR)-medicarpin.^51,53,83^

Immunolocalization studies showed that vestitone reductase (VR) and isoflavone reductase (IFR) are cytosolic enzymes. Notably, VR accumulates in a narrow zone of leaf cells around fungal lesions, in all cell types of both mature and young roots, and in the nodule meristem as well as a lateral nodular tissues.^57^ In *Cicer arietinum*, elicitation with yeast glucan revealed that low to moderate concentrations of the elicitor stimulate pterocarpan conjugate production via the action of glucosyl and malonyltransferases, which can then be stored in vacuoles. At higher elicitor concentrations, the aglycone form accumulates as extracellular phytoalexins, driven by increased activity of enzymes such as PAL, C4H, and CHS.^46^

In vitro assays showed that the ABCG10 protein in *Medicago truncatula* facilitates the translocation of key intermediates in the phenylpropanoid pathway, including 4-coumarate and liquiritigenin. Silencing *MtABCG10* expression reduces medicarpin accumulation; however, the biosynthesis can be partially restored with exogenous 4-coumarate and formononetin.^62^

### 4.3 Extraction and Isolation Techniques

In recent years, significant advancements have been made in the extraction and isolation techniques for medicarpin. However, methanol and ethanol remain the most commonly employed primary solvents for extracting medicarpin from plant materials, primarily due to their efficacy, as demonstrated in the *Medicago sativa* model.^56^ In this species, early studies established foundational methods for isolating medicarpin from roots, where this phytoalexin is particularly concentrated. For instance, medicarpin-β-d-glucoside was isolated by preparing a methanol extract, which was then subjected to charcoal chromatography. Following elution and purification, medicarpin was derived from this glycoside through enzymatic hydrolysis using a commercial crude enzyme obtained from snail digestive fluid.^55^

These advancements, coupled with an improved understanding of medicarpin’s chemical structure, led to modifications in extraction protocols. Solvents of lower to medium polarity were used for initial extraction, followed by identification or isolation via thin-layer chromatography (TLC). For example, in *Vicia faba* infected with *Botrytis cinerea*, diethyl ether was utilized for extraction.^68^ Similar protocols were applied to fungal-infected tissues of *Trifolium pratense* and fungal diffusates, where carbon tetrachloride and ethanol were used (6). In rotted cotyledons of *Canavalia ensiformis* infected with *Rhizoctonia solani* mycelium, chloroform served as the extraction solvente.^44,48^

As chromatography technology advanced, TLC was combined with additional purification techniques. For instance, chromatography on LH-20 Sephadex was used for purifying medicarpin from the leaves of *Sophora japonica* inoculated with *Helminthosporium carbonum*.^63^ This approach was later applied to fungus-infested seedlings of *Trigonella foenum-graecum* to separate traces of maackiain, another common pterocarpan phytoalexin.^67^

Subsequent research has refined these methods, incorporating novel techniques such as high-performance liquid chromatography (HPLC). For instance, medicarpin was purified from a chloroform extract of *Dalbergia odorifera* through initial elution on a Sephadex LH-20 chromatography column, followed by preparative HPLC on a reverse phase (RP) ODS-2 C18 column.^16^ There has been a continued improvement in extraction and metabolite isolation techniques. *M*. *sativa* foliage was extracted with ethanol and fractionated on a silica column using a gradient of hexane, ethyl acetate, and methanol. Preparative silica TLC with methanol-chloroform was employed to purify medicarpin for HPLC gradient chromatography.^59^ Other stationary phases were explored, such as polyamide column chromatography followed by preparative HPLC with RP-18 columns to isolate medicarpin 3-O-glucoside from cold acetone and methanol extracts of *Cicer arietinum* cells.^47^

Olther researchers investigated various induction and extraction techniques for medicarpin production. For example, copper-induced production in *T*. *foenum-graecum* cell cultures was extracted using methanol and ultrasonication, with the final isolation achieved through semipreparative HPLC with an RP-18 column.^11^ Ultrasonication was also applied to the extraction of medicarpin and other metabolites from *Glycyrrhiza uralensis* and *Glycyrrhiza glabra*, where a 70% methanol extract was obtained from root poder.^50^ In simpler extraction methods, spontaneous crystallization of medicarpin was observed during Soxhlet extraction with hexane from *Dalbergia congestiflora* milled wood, where medicarpin crystallized on the upper walls of the flask.^14^

Additional procedures have incorporated normal-phase silica gel (200–300 mesh) and reversed-phase C18 silica gel column chromatography, as in the isolation of medicarpin from *Maackia amurensis*.^37^ Silica gel and HPLC have been complemented by medium-pressure liquid chromatography (MPLC) with reverse-phase technology, as used in the isolation of medicarpin from *Homalomena polybotrys* roots^23^ and *Crotalaria lineata* pods.^25^ Bioassay-guided fractionation was also integrated into isolation processes, as shown by Williams et al.,^15^ where flash chromatography facilitated the extraction and isolation of medicarpin from Jamaican multifloral propolis.

In conclusion, methodologies for isolating medicarpin have progressed from basic solvent extractions and TLC to sophisticated multistep processes incorporating liquid–liquid partitioning, ultrasound-assisted extraction, classical columns, MPLC, flash chromatography, and HPLC. These advancements not only enhance the reproducibility and yield of high-purity medicarpin but also support precise quantification and bioactivity assessment. Differences in the extraction and isolation methods, including commonly used solvents, along with their respective advantages and disadvantages are presented and discussed in Table A (Supporting Information).

### 4.4 Medicarpin Synthesis

The synthesis of various pterocarpans, including racemic (±)-medicarpin, commonly involves a key borohydride reductive cyclization step to form the pterocarpan four-ring system through a three-step pathway (Figure 4). Initially, a mixture of resorcinol and 2,4-dimethoxyphenyl acetic acid stirred with the Lewis acid BF3-OEt_2_ at 90–100 °C yields a deoxybenzoin derivative.^31,36^ This intermediate, upon treatment with mesyl chloride in dimethylformamide (DMF), produces 2′-methoxyformononetin (2MF). Selective demethylation of 2MF can be achieved using AlCl_3_ and acetonitrile under reflux conditions,^13,28,33,84^ resulting in 2′-hydroxyformononetin (2HF). Subsequent reduction of 2HF with sodium borohydride in ethanol yields medicarpin, which can be recrystallized from ethyl acetate in hexane.^29,36^

**Figure 4:**
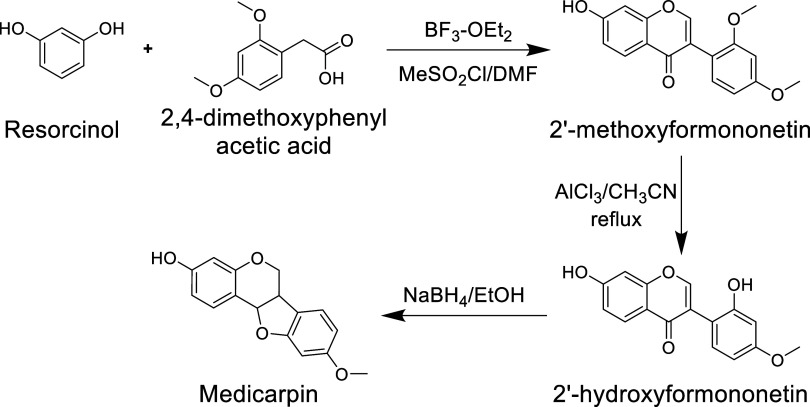
Synthesis of medicarpin via borohydride reductive cyclization. **BF**_**3**_ **-OEt**_**2**_: boron trifluoride-ether; **MeSO**_**2**_: methanesulfonyl chloride; **DMF**: dimethylformamide; **AlCl**_**3**_: aluminum chloride; **CH**_**3**_ **CN**: acetonitrile; **NaBH**_**4**_: sodium borohydride.

Several enantioselective synthesis strategies have been explored. One method involves a nine-step total synthesis of (−)-medicarpin, achieving a 4% overall yield. The key diastereoselective step employs a nonracemic alcohol, combined with benzaldehyde and sodium hexamethyldisilazide, to produce an ortho-quinone via a methide Diels–Alder reaction.^85^ This is followed by oxidative cyclization through a para-quinone intermediate. Another approach focuses on synthesizing (+)-medicarpin through an asymmetric Evans’ aldol reaction, setting the required stereochemistry at positions C6a and C11a.^86^ Starting from 4-benzyloxy-2-hydroxybenzaldehyde and 4-methoxy-2-hydroxybenzaldehyde, intermediates are prepared and modified via Wittig olefination, hydrolysis, and Pinnick oxidation. The product is then converted with a chiral oxazolidinone, forming the aldol adduct with precise stereochemical control. A series of protection and deprotection steps, followed by Mitsunobu reaction, forms the B-ring into a chromane structure. Final regioselective palladium-catalyzed hydrogenation and cyclization complete the (+)-medicarpin synthesis, yielding an 11-step procedure with an overall yield of 11%.

### 4.5 Analytical Methods for Medicarpin Identification and Quantification

Initial attempts to identify medicarpin relied on a combination of spectroscopic techniques, including Nuclear Magnetic Resonance (NMR), Infrared Spectroscopy (IR), Mass Spectrometry (MS), Ultraviolet (UV) spectroscopy, and Optical Rotatory Dispersion (ORD). These methods were used to elucidate medicarpin-β-d-glucoside from *Medicago sativa* ^55^ and its aglycone form from *Vicia faba* (68). Early studies also employed thin-layer chromatography (TLC) on silica gel plates to analyze phytoalexins, using low to moderately polar mobile phases such as propanol-ethyl acetate-water,^69^ chloroform-carbon tetrachloride, hexane-ethyl acetate-methanol,^54^ ether-ethyl acetate-methanol,^64^ chloroform–methanol, and *n*-pentane-diethyl ether-glacial acetic acid.^42^ However, these approaches were somewhat limited, relying on UV fluorescence for analysis^52,54^ or spot identification using retention factors and color reactions with p-nitroaniline reagente.^6,42,54^

As research progressed, methods for quantifying medicarpin in plants were developed. In some cases, after TLC analysis, the silica gel spots containing medicarpin were extracted and analyzed spectrophotometrically (250–320 nm), with compound confirmation via UV and MS.^54^ Weltring and Barz^80^ used UV spectra at 287 nm to study the degradation of medicarpin by *Fusarium proliferatum*, later refining this approach to quantify the compound at 285 nm and adding MS for confirmation.^78^ Other quantification methods involved gas chromatography (GC) using pyridine-treated samples^44,48^ or gas chromatography–mass spectrometry (GC-MS) in selective ion monitoring mode to identify medicarpin and its biosynthetic precursors in *Trifolium repens*.^64^

With advancements in high-performance liquid chromatography (HPLC), both enantiomers of medicarpin were identified in *Sophora japonica* leaves inoculated with *Helminthosporium carbonum* using HPLC, UV, and MS.^63^ Medicarpin was further isolated from *M*. *sativa* and *C*. *ensiformis* cotyledons and purified using HPLC with porous silica columns such as μPorasil.^43^ Extracted medicarpin from *Arachis hypogaea* leaflets naturally infected with fungi was quantified by HPLC with an internal standard.^42^ Over time, the combination of TLC, UV–visible absorption spectra, HPLC, and GC-MS became more prevalent in analyzing medicarpin and its catabolites.^77^

Miller et al.^16^ used both analytical and preparative HPLC with ODS C18 columns to isolate medicarpin from *Dalbergia odorifera*, identifying it through ^1^H NMR and MS. However, this method had limitations, as it could not distinguish between medicarpin and isomedicarpin. Later, Guo, Dixon, and Paiva^53^ employed semipreparative HPLC to purify medicarpin from *M*. *sativa*, utilizing reverse-phase HPLC, UV, MS, and Circular Dichroism (CD) for identification. This combination of techniques remains widely used, not only for identifying medicarpin but also for verifying its purity through HPLC with UV detection at 280 nm.^37^

Advancements include the use of capillary electrophoresis (CE) to separate enantiomers of medicarpin and vestitone, employing optimized running electrolytes and achieving rapid analysis within 12 min.^41^ The development of HPLC methods continues to play a significant role in medicarpin quantification, often involving calibration curves and comparisons with medicarpin standards.^56^ For example, reverse-phase columns like Lichrosorb RP 18 have been used to analyze medicarpin in *T*. *foenum-graecum* roots under metal exposure, with quantification based on calibration curves.^10^

The most advanced approaches have led to the development and validation of rapid methods for quantifying medicarpin in biological samples. Taneja et al.^35^ developed a 3 min HPLC method for quantifying medicarpin in rat plasma using an X-Bridge RP18 column coupled to a quadrupole ion trap mass spectrometer (QTRAP), equipped with electrospray ionization (ESI) in multiple reaction monitoring (MRM) mode. This method was later optimized for studies of medicarpin bioavailability, tissue distribution, and excretion in various biological matrices.^36^

Recent work by Sharma et al.^31^ involved synthesizing medicarpin and identifying it via HPLC using an X-Bridge C18 column, alongside ^1^H NMR, ^13^C NMR, and HRMS (ESI). Meanwhile, Williams et al.^15^ identified the enantiomer (+)-medicarpin from Jamaican multifloral propolis using LC-MS with a reversed-phase analytical column, ^1^D and ^2^D NMR analysis for ^1^H and ^13^C, and optical rotation. Finally, modern hyphenated methods like UPLC-QTOF/MS are being employed to analyze medicarpin. For example, *C*. *lineata* was analyzed using UPLC-QTOF/MS with ESI and NMR spectroscopy to identify mass fragmentation patterns.^25^ Additionally, UPLC-ESI-QTOF-MS/MS has been used to study biosynthetic pathways and quantify medicarpin yield in *Saccharomyces cerevisiae*.^50^ In 2021, Aldana-Mejía and collaborators reported an HPLC-UV method for the detection and quantification of phenolic compounds, including medicarpin, in Brazilian red propolis—a complex matrix of compounds—and in *D*. *ecastaphyllum*.

While significant progress has been made in identifying and quantifying medicarpin, challenges remain, particularly in distinguishing between isomers and accurately quantifying low concentrations in complex biological matrices. The reliance on UV and MS data for confirmation, while standard, may not always provide sufficient specificity, especially in distinguishing isomers or enantiomers. Furthermore, the development of faster, more sensitive methods, such as UPLC and HPLC-MS/MS, has improved analysis but may still require further optimization for broader applications in various matrices. Differences in the identification and quantification methods, along with their respective advantages and disadvantages are arranged and discussed in Table B (Supporting Information). Despite these challenges, the continued evolution of analytical techniques holds promise for advancing our understanding of medicarpin’s role in plant defense and its potential therapeutic applications.

## 5 *In* *Vitro* Evaluations and Cell Culture Models

### 5.1 Medicarpin and Bone Metabolism

Medicarpin has garnered attention for its potential benefits in bone health, primarily due to its dual role in promoting osteoblast differentiation and inhibiting osteoclastogenesis. In vitro studies have demonstrated that medicarpin enhances osteoblast differentiation by activating key signaling pathways^87^ while also inhibiting the formation and function of osteoclasts, which contributes to its antiresorptive Properties. ^33^Both medicarpin and its derivatives have shown the ability to suppress osteoclastogenesis, supporting their potential in maintaining bone homeostasis by simultaneously promoting bone formation and inhibiting bone resorption.^30^ Furthermore, medicarpin has exhibited anti-inflammatory and apoptosis-inhibiting effects,^87^ and has been found to regulate immune responses by mitigating TNF-α-induced T cell senescence.^34^ Its selective activation of estrogen receptor β (ERβ) with minimal estrogenic effects in nonskeletal tissues suggests its potential as a therapeutic agent for osteoporosis. However, further studies are necessary to assess its safety profile in comparison to traditional estrogen therapies.^28^

The ability of medicarpin to stimulate osteoblast differentiation and matrix mineralization has been demonstrated through the upregulation of key osteogenic markers, such as alkaline phosphatase (ALP), runt-related transcription factor 2 (Runx-2), and bone morphogenetic protein-2 (BMP-2). These effects are primarily mediated by the activation of the BMP-2 and p38 MAPK signaling pathways.^87^ Similarly, 9-demethoxy-medicarpin (DMM) promotes osteoblast differentiation and mineralization through the same mechanisms, enhancing the expression of Runx-2 and Osterix.^30^

The antiresorptive properties of medicarpin are evident in its ability to inhibit osteoclast formation and activity. It inhibited the differentiation of osteoclast precursors in a dose-dependent manner, independent of estrogen receptor activation.^33^ Furthermore, medicarpin modulates the osteoprotegerin (OPG)/receptor activator of nuclear factor kappa-B ligand (RANKL) ratio, a key regulator of osteoclastogenesis. DMM also suppresses osteoclast formation through similar modulation of the OPG/RANKL ratio.^30^ This dual action, promoting osteoblast activity while inhibiting osteoclastogenesis, supports medicarpin’s role in maintaining bone homeostasis (Figure 5).

**Figure 5:**
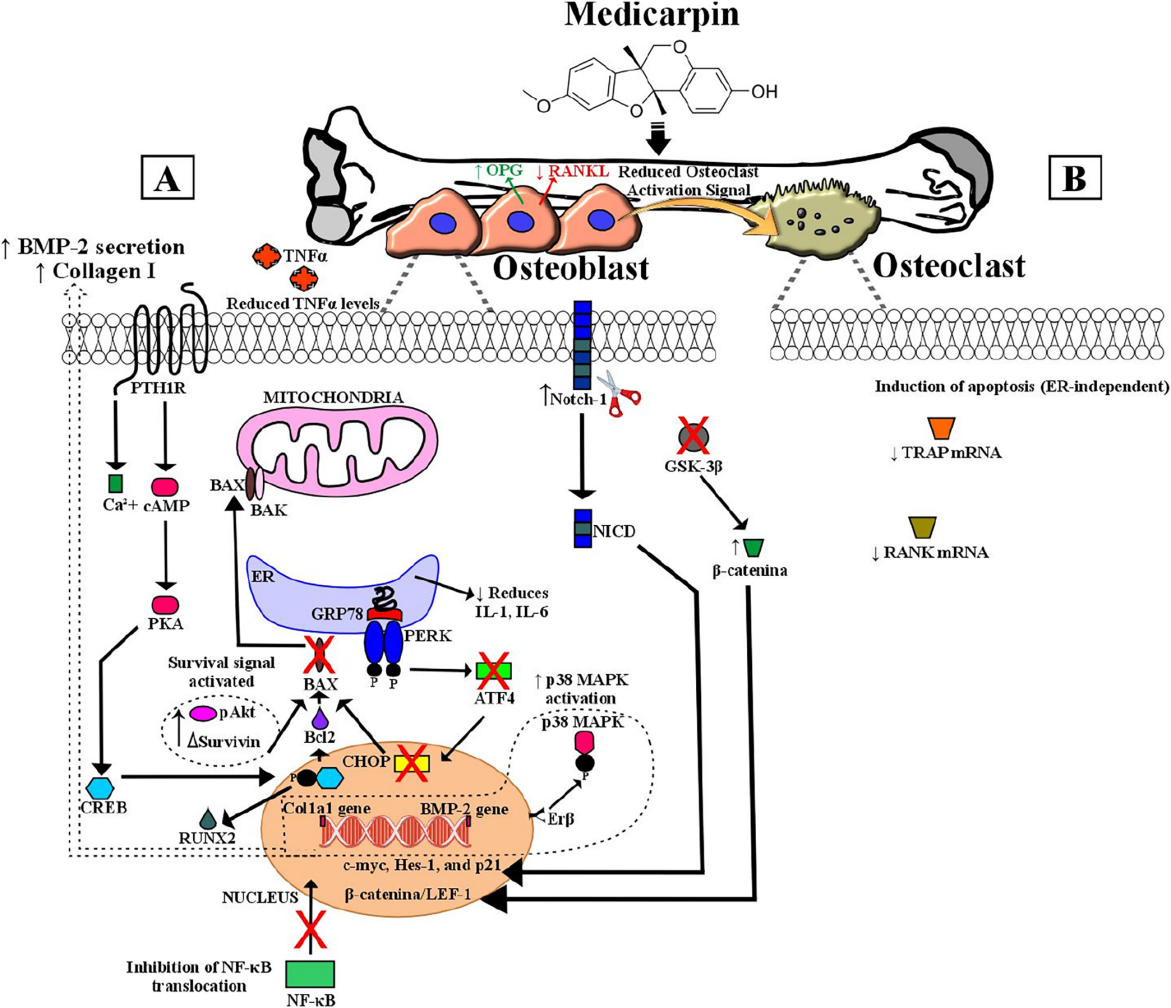
Mechanisms of action of medicarpin on bone homeostasis. **Abbreviations**: BMP-2, Bone Morphogenetic Protein-2; ERβ, Estrogen Receptor β; GSK-3β, Glycogen Synthase Kinase-3β; GRP78, Glucose-Regulated Protein 78; NF-κB, Nuclear Factor Kappa B; NICD, Notch Intracellular Domain; OPG, Osteoprotegerin; p38 MAPK, p38 Mitogen-Activated Protein Kinase; PTH1R, Parathyroid Hormone 1 Receptor; RANK, Receptor Activator of Nuclear Factor Kappa-B; RANKL, RANK Ligand; TNF-α, Tumor Necrosis Factor-α; TRAP, Tartrate-Resistant Acid Phosphatase. Arrows (→) indicate activation; symbols (X) indicate inhibition.

Medicarpin promotes bone formation by osteoblasts (Figure 5A) and inhibits bone resorption by osteoclasts (Figure 5B) through multiple signaling pathways and paracrine interactions. (Figure 5A) **Osteoblasts**: Medicarpin activates ERβ and inhibits GSK-3β, stimulating Wnt/β-catenin, Notch, and p38 MAPK pathways. This enhances BMP-2 and Collagen I secretion, increases antiapoptotic factors (pAkt, BCL-2, survivin), reduces ER stress (GRP78-PERK-CHOP), inhibits NF-κB-mediated inflammation, and potentiates PTH1R-cAMP-PKA-CREB signaling. (Figure 5B) **Osteoclasts:** Medicarpin induces apoptosis in mature osteoclasts and indirectly inhibits osteoclastogenesis by increasing the OPG/RANKL ratio from osteoblasts.

Bone regeneration is further enhanced by medicarpin through the activation of critical signaling pathways. Dixit et al.^29^ demonstrated that medicarpin activates both the Wnt and Notch pathways in preosteoblasts, which are essential for osteoblast differentiation. This activation leads to an increase in β-catenin and lymphoid enhancer-binding factor 1 (LEF-1) expression, while simultaneously inhibiting glycogen synthase kinase-3 β (GSK-3β), a negative regulator of the Wnt pathway. Moreover, medicarpin upregulates Notch-1 and Jagged-1, along with their target genes such as c-myc, Hes-1, and p21, further promoting osteoblast differentiation.

The osteogenic effects of medicarpin have also been linked to its selective activation of estrogen receptor β (ERβ). Studies by Bhargavan et al.^28^ showed that medicarpin enhances osteoblast differentiation without inducing estrogenic effects in nonskeletal tissues, such as the uterus. This selective activation of ERβ suggests that medicarpin could be a potential therapeutic candidate for osteoporosis, reducing the risks associated with traditional estrogen therapies. Table 2 presents the main *in vitro* effects of medicarpin and its derivatives on bone metabolism.

**Table 2:** *In vitro* Effects of Medicarpin and Derivatives on Osteoblast Differentiation, Mineralization and Osteoclastogenesis^a^

substance	model	assay	dose/concentration	effect	mechanism^a^	references
9-demethoxy-medicarpin (DMM)	Rat calvarial osteoblasts, mouse bone marrow cells	ALP activity, mineralization (Alizarin Red-S), TRAP assay, NF-κB translocation	100 pM (osteoblast differentiation and mineralization), 100 pM–10 nM (osteoclastogenesis)	Increased osteoblast differentiation and mineralization, decreased osteoclast formation	Activation of BMP-2 and P38MAPK pathways, increased OPG/RANKL ratio, inhibition of RANKL signaling	^30^
Medicarpin	Calvarial osteoblasts (Neonatal Mice)	2D Gel Electrophoresis, qPCR, Western Blotting, Annexin-PI Staining	10 nM Medicarpin	Downregulation of Glucose-Regulated Protein 78, inhibition of ER stress-induced apoptosis, increased osteoblast survival by preventing excessive ER stress.	Inhibition of mitochondrial-mediated apoptosis, downregulation of pro-apoptotic factors (ATF4, CHOP, Bax), increased antiapoptotic factors (survivin, pAkt), attenuation of ER stress by blocking the CHOP pathway.	^87^
	Mouse calvarial osteoblasts	Cell viability assay (MTT), mineralization assay (Alizarin Red), intracellular calcium assay (Fluo-3-AM)	Parathyroid Hormone PTH (100 nM) + Medicarpin (100 pM)	Enhanced osteoblast viability, increased mineralization, elevated intracellular calcium concentration improved osteoblast proliferation when combined with PTH.	Augmentation of cAMP - PKA - CREB pathway, increased expression of RUNX2 and BCL-2, inhibition of apoptosis via PKA signaling, enhancement of CREB phosphorylation	^31^
Medicarpin	Calvarial osteoblasts	NF-κB translocation assay, qPCR	10^–10^ M medicarpin	Inhibition of TNFα-induced NF-κB translocation, reduced TNFα mRNA levels	ER-dependent reduction in pro-inflammatory cytokines (IL-1, IL-6)	^33^
	Rabbit osteoclasts	Apoptosis assay (Hoechst staining)	10^–10^ M to 10^–8^ M medicarpin	Induced apoptosis in mature osteoclasts	Estrogen receptor-independent mechanism, inhibition of osteoclast survival	
	Coculture of osteoblasts and bone marrow cells	qPCR analysis of gene expression	10^–10^ M medicarpin	Increased OPG/RANKL ratio, reduced TRAP and RANK mRNA levels	ER-independent effect in osteoblasts, enhancing OPG expression and reducing osteoclastogenic signals	
Medicarpin	Rat calvarial osteoblasts	ALP activity assay, Mineralization assay (Alizarin Red-S)	10 nM Medicarpin	Increased osteoblast differentiation and mineralization, associated with increased expression of osteogenic markers such as Col1a1.	Activation of p38 MAPK pathway, increased BMP-2 secretion, and Erβ-dependent signaling	^28^
	COS-7 Cells	ER transactivation assay, coactivator interaction assay	10 nM Medicarpin	Enhanced ERβ transactivation and interaction with coactivators	ER and p38 MAPK signaling pathways, promoting osteogenic gene expression	
	HepG2 Cells	BMP receptor activity assay	10 nM Medicarpin	No direct BMP receptor agonism	Not a direct BMP receptor agonist	

a **Abbreviation**: **ALP:** Alkaline Phosphatase. **TRAP:** Tartrate Resistant Acid Phosphatase. **NF-κB:** Nuclear Factor Kappa B. **BMP-2:** Bone Morphogenetic Protein **2**. **P38MAPK:** P38 Mitogen-Activated Protein Kinase. **OPG:** Osteoprotegerin. **RANKL:** Receptor Activator of Nuclear Factor Kappa-B Ligand. **qPCR:** Quantitative Polymerase Chain Reaction. **ER:** Endoplasmic Reticulum. **PI:** Propidium Iodide. **PTH:** Parathyroid Hormone. **cAMP:** Cyclic Adenosine Monophosphate. **PKA:** Protein Kinase A. **CREB:** Cyclic AMP Response Element-Binding Protein. **RUNX2:** Runt-Related Transcription Factor 2. **BCL-2:** B-cell Lymphoma 2. **TNFα:** Tumor Necrosis Factor α. **IL-1:** Interleukin-1. **IL-6:** Interleukin-6. **ATF4:** Activating Transcription Factor 4. **CHOP:** C/EBP Homologous Protein. **Bax:** Bcl-2-associated X protein. **MTT:** Methylthiazolyldiphenyl-tetrazolium bromide. **Fluo-3-AM:** Fluorescent dye used to measure intracellular calcium. **Col1a1:** Collagen Type I α 1 Chain. **ERβ:** Estrogen Receptor β.

While the ability to modulate osteoblast and osteoclast function represents a shared characteristic among various phytoestrogens, including genistein and daidzein, medicarpin distinguishes itself through its superior potency. Evidence demonstrates that medicarpin stimulates osteoblast differentiation and inhibits osteoclastogenesis at nanomolar (nM) to picomolar (10^–10^ M) concentrations, whereas most other reported flavonoids require micromolar (μM) concentrations to achieve comparable effects.^28,33^ This enhanced bioactivity likely stems from its methoxylated isoflavonoid structure, a distinctive feature that has been consistently associated with increased osteogenic potency among compounds within this class.^28^ Consequently, while the broader mechanistic framework may be conserved across flavonoids, medicarpin’s potency profile, which can be directly attributed to its chemical architecture, establishes it as a compound with considerable therapeutic potential.

### 5.2 Anti-Inflammatory and Apoptosis-Inhibiting Effects

In addition to its osteogenic and antiresorptive effects, medicarpin exhibits anti-inflammatory and apoptosis-inhibiting properties. It reduces pro-inflammatory cytokine expression, such as interleukin-1 (IL-1) and interleukin-6 (IL-6), by inhibiting TNF-α-induced nuclear translocation of NF-κB in osteoblasts, which contributes to a reduction in osteoclastogenesis and fosters an environment favorable for osteoblast differentiation.^87^ Medicarpin also prevents endoplasmic reticulum (ER) stress-induced apoptosis by downregulating glucose-regulated protein 78 (GRP78) in osteoblasts. Additionally, it inhibits mitochondrial apoptosis by reducing cytochrome c release and caspase-3 activity, while increasing survivin levels, thus promoting osteoblast survival.^87^

Medicarpin was identified as a potent leukotriene inhibitor in AB-CXBG mouse mastocytoma cells, with an IC_50_ of 0.52 μg/mL, demonstrating its effectiveness in blocking inflammatory pathways.^16^ This compound selectively inhibits the 5-lipoxygenase pathway without affecting cyclooxygenase, highlighting its specificity as an anti-inflammatory agent. Additionally, another study showed that medicarpin modulates immune responses by reducing the proportion of IL-17A + TH-17 cells and increasing FoxP3 + Treg cells, which are crucial for controlling inflammation and osteoclastogenesis in arthritis models.^84^

### 5.3 Protection Against Premature T Cell Senescence

Medicarpin’s role in immune regulation was highlighted in studies focused on T cell senescence. Tyagi et al.^34^ demonstrated that medicarpin prevents TNF-α-induced T cell senescence in bone marrow-derived T cells by preserving CD28 expression and reducing reactive oxygen species (ROS) levels. By mitigating oxidative stress and inflammatory responses, medicarpin helps maintain bone health by regulating immune cell activity within the bone microenvironment.

### 5.4 Neuroprotective and Anti-Neurodegenerative Effects

Medicarpin exhibits significant neuroprotective and antineurodegenerative effects by modulating cholinergic function, inhibiting monoamine oxidase-B (MAO-B), and reducing both apoptosis and inflammation. These actions position medicarpin as a potential therapeutic candidate for neurodegenerative diseases such as Alzheimer’s and Parkinson’s by targeting key pathways essential for neuronal survival and synaptic plasticity. However, further studies are necessary to fully evaluate its therapeutic efficacy.

In Alzheimer’s disease models, medicarpin inhibits acetylcholinesterase (AChE), which helps maintain acetylcholine levels, thereby enhancing cognitive function. Additionally, it upregulates brain-derived neurotrophic factor (BDNF) and phosphorylated CREB (p-CREB), promoting neurogenesis and synaptic maintenance, both critical for memory and learning processes.^88,25^

In Parkinson’s disease models, medicarpin selectively inhibits MAO-B, preserving dopamine levels and mitigating motor and cognitive deficits. Furthermore, this inhibition reduces the production of hydrogen peroxide, lowering oxidative stress and providing neuroprotection to dopaminergic neurons.^25^

Medicarpin also demonstrates significant antiapoptotic and anti-inflammatory effects. It inhibits pro-apoptotic proteins such as caspase-3 while enhancing antiapoptotic factors like Bcl-2, a process regulated by the activation of the PI3K/Akt pathway, promoting cell survival. In microglial cells, medicarpin reduces nitric oxide (NO) production, a marker of neuroinflammation, which is critical for minimizing neuronal damage in diseases like Alzheimer’s and Parkinson’s.^23^

In the context of neuroinflammation, medicarpin effectively modulates microglial polarization. In a model of chronic unpredictable mild stress (CUMS)-induced depression, medicarpin reduced pro-inflammatory M1 microglia while promoting anti-inflammatory M2 microglia in the amygdala. At a dosage of 5 mg/kg, it downregulated pro-inflammatory markers, such as iNOS and CD16, and increased the expression of anti-inflammatory markers like Arg-1 and CD206, thereby confirming its role in suppressing neuroinflammation through LXRβ modulation.^24^

Moreover, medicarpin enhances synaptic plasticity by upregulating BDNF and CREB, supporting neurogenesis and synaptic development. These mechanisms are crucial for cognitive function, especially in conditions associated with synaptic loss.^88^

Under ischemic conditions, medicarpin protects cerebral endothelial cells by activating the PI3K/Akt/FoxO pathway. This activation boosts antioxidant enzyme activity, reduces oxidative stress, and inhibits apoptosis. By protecting the blood-brain barrier and mitigating endothelial damage, medicarpin may help reduce the impact of ischemic stroke.^27^

### 5.5 Antiproliferative and Pro-apoptotic Effects of Medicarpin in Cancer Cells

Medicarpin has demonstrated significant antiproliferative and pro-apoptotic effects in various in vitro cancer models by targeting key apoptotic and survival pathways. It induces apoptosis through both intrinsic and extrinsic mechanisms, enhances sensitivity to chemotherapeutic agents, and modulates crucial signaling pathways such as PTEN/AKT, highlighting its potential as a versatile therapeutic agent.

Studies on leukemia models showed that medicarpin was effective in overcoming multidrug resistance. In murine P388 leukemia cells, including doxorubicin-resistant variants (P388/DOX), medicarpin significantly reduced cell viability (IC_50_ = 90 μM). The apoptosis triggered by medicarpin involved the mitochondrial pathway, altering the balance of pro-apoptotic (Bax, Bak) and antiapoptotic (Bcl-2, Bcl-XL) proteins. Moreover, medicarpin enhanced the efficacy of doxorubicin and vinblastine by promoting drug uptake in resistant cells.^19^

In bladder cancer cells, such as T24 and EJ-1, medicarpin induced G1 phase cell cycle arrest and promoted apoptosis via the mitochondrial pathway. This was accompanied by the upregulation of pro-apoptotic proteins, including BAK1, Bcl2-L-11, and caspase-3, as well as a reduction in cell proliferation (IC_50_ = 65 μM).^18^

Medicarpin was also active in glioblastoma models, where it suppressed cell proliferation and triggered apoptosis through both intrinsic and extrinsic pathways. In U251 and U-87 MG cells, medicarpin caused G2/M phase arrest and increased the expression of BID, BAX, CASP3, CASP8, and CYCS, with IC_50_ values of 154 μg/mL and 161 μg/mL, respectively. Additionally, it reduced cell migration, further supporting its potential as a therapy for glioblastoma.^22^

In myeloid leukemia, medicarpin sensitized cells to TRAIL-induced apoptosis by upregulating the death receptor DR5 through the ROS-JNK-CHOP pathway. The combination of medicarpin and TRAIL significantly enhanced apoptosis by activating both the extrinsic (caspase-8) and intrinsic (caspase-9) pathways. Medicarpin also modulated apoptotic proteins, downregulating antiapoptotic factors (Bcl-2, XIAP, survivin) and upregulating pro-apoptotic proteins (Bax, cytochrome C, tBid), suggesting its potential as a sensitizer in combination therapies.^20^

Lastly, in head and neck squamous cell carcinoma (HNSCC), medicarpin modulated the PTEN/AKT signaling pathway by increasing PTEN and AKT expression while reducing PDK1 expression. These effects resulted in decreased cell viability (IC_50_ = 80 μM), with Western blot analysis confirming enhanced PTEN and AKT phosphorylation and reduced PDK1 phosphorylation. This indicates that medicarpin may inhibit cancer progression by modulating the PI3K/AKT/mTOR pathway, with potential applications in combination treatments for HNSCC.^21^

### 5.6 Antimicrobial Activity

Medicarpin completely inhibited the growth of *Trametes versicolor* at a MIC of 150 μg/mL, demonstrating its effectiveness in preventing fungal decay.^14^ Similarly, medicarpin and its biosynthetic precursors were tested against fungal pathogens of alfalfa (*Medicago sativa*), including *Phytophthora megasperma* and *Phoma medicaginis*. Medicarpin showed significant inhibition of *P*. *megasperma* at concentrations as low as 0.5 mM, with intermediates like vestitone and 2′-hydroxyformononetin also displaying antifungal activity, although medicarpin remained the most potente.^12^ Duczek and Higgins^6^ demonstrated that medicarpin inhibited oxygen uptake and germ tube growth in *Helminthosporium carbonum* and *Stemphylium botryosum*, highlighting its role in red clover’s defense by preventing *H*. *carbonum* infection. Additionally, in a study on Jamaican propolis, medicarpin selectively inhibited *Neisseria gonorrheae* with a MIC of 250 μg/mL, while showing no activity against *Staphylococcus aureus* or *Escherichia coli*. When combined with vancomycin, medicarpin had an additive effect, lowering the concentration needed to inhibit bacterial growth.^15^

### 5.7 Metabolic Effects: Browning and Lipolysis via AMPK and PKA Pathways

In studies using C3H10T1/2 mesenchymal stem cells, medicarpin demonstrated remarkable efficacy in inducing the browning of white adipocytes at an effective concentration of 10 μM. By activating the AMP-activated protein kinase (AMPK) pathway, medicarpin upregulated key thermogenic markers, including uncoupling protein 1 (UCP1), Prdm16, Pgc-1α, and Pparγ, while downregulating white adipocyte markers such as Leptin and Serpina3k. This highlights its potential in metabolic regulation and energy expenditure. Additionally, at the same concentration, medicarpin enhanced mitochondrial biogenesis by increasing the expression of mitochondrial genes such as Cox7a, Cox8b, and Tfam, further supporting its role in metabolic regulation.^89^

Another study focusing on brown adipose tissue (BAT) revealed that medicarpin at a concentration of 10 μM promoted lipolysis. At this concentration, medicarpin induced glycerol release and upregulated lipolytic enzymes, including hormone-sensitive lipase (HSL) and adipose triglyceride lipase (ATGL). The compound facilitated the phosphorylation of HSL at serine 660, a crucial step in activating lipolysis. This, in turn, increased fatty acid oxidation, reinforcing medicarpin’s potential to enhance energy production and fat mobilization.^89^

### 5.8 Additional Biological Activities of Medicarpin

Medicarpin exhibits a range of biological activities across different systems, demonstrating its versatility as a bioactive compound. Medicarpin exhibited potent antioxidant activity in vitro. In HeLa cells, it enhances NRF2 translocation to the nucleus and upregulates antioxidant response element (ARE) activity. At a concentration of 50 μM, medicarpin significantly increased the expression of key antioxidant genes, including HO-1, GCLC, and NQO-1. Additionally, it inhibited NRF2 proteasomal degradation, leading to increased stabilization and an enhanced antioxidant response. However, at higher concentrations, such as 100 μM, medicarpin inhibited cell growth.^17^ Table 3 presents the main biological activities evaluated in vitro regarding the effects of medicarpin and its derivatives.

**Table 3:** Biological Activities Evaluated *In vitro* on The Effects of Medicarpin and Its Derivatives^a^

activities	substance	model	assay	dose/concentration	effect	mechanism	references
Neuroprotective and Anti-Neurodegenerative	Medicarpin	Microglial cells (BV2)	Nitric oxide production in LPS-stimulated cells	IC_50_ = 5 ± 1 mM	Reduced NO production, anti-inflammatory	Anti-inflammatory activity via NO reduction	^23^
		Neuronal cells (N2A) under OGD	Apoptosis reduction under oxygen-glucose deprivation	IC_50_ = 13 ± 2 mM	Reduced apoptosis, antiapoptotic effects	Inhibition of caspase-3, increased Bcl-2 levels	
		Human cerebral microvascular endothelial cells (HCMECs)	Cell viability, oxidative stress, and apoptosis assays	20 and 30 μM	Improved cell viability, reduced oxidative stress and apoptosis	Activation of PI3K/Akt/FoxO pathway	^27^
		Human monoamine oxidase-B (MAO-B) inhibition assay	Enzyme inhibition assay	IC_50_ = 0.45 μM	Potent inhibition of MAO-B with minimal activity against MAO-A	Competitive reversible inhibition of MAO-B	^25^
		SH-SY5Y human neuroblastoma cells	AChE inhibition assay, Western blot analysis for BDNF and p-CREB expression	3 and 10 μM	Decreased AChE activity, increased BDNF and p-CREB expression	Cholinergic enhancement through AChE inhibition, neuroprotection via BDNF and CREB upregulation	
		Molecular docking of medicarpin with MAO-B	In silico molecular docking study	N/A (in silico study)	Strong binding affinity to MAO-B active site	Predicted competitive binding of medicarpin to the MAO-B active site, supporting inhibition	
Anticancer Activity	Medicarpin	Leukemia (P388/DOX)	Cell Viability (MTT)	IC_50_ = 90 μM	Reduced cell viability, sensitized to doxorubicin	Mitochondrial apoptosis, enhanced drug uptake	^19^
		Bladder Cancer (T24, EJ-1)	Flow Cytometry	IC_50_ = 65 μM	G1 phase cell cycle arrest, apoptosis	Mitochondrial apoptosis, upregulation of BAK1, Bcl2-L-11, caspase-3	^18^
		Glioblastoma (U251, U-87 MG)	Cell Viability (MTT), Migration Assay	IC_50_ was 154 μg/mL for U251 cells and 161 μg/mL for U-87 MG cells	G2/M phase cell cycle arrest, reduced migration	Upregulation of BID, BAX, CASP3, CASP8, CYCS	^22^
		Myeloid Leukemia	Cell Viability (CCK-8), Apoptosis Assay	20 μM	Enhanced TRAIL-induced apoptosis	Upregulation of DR5, activation of ROS-JNK-CHOP pathway	^20^
		Head and Neck Squamous Cell Carcinoma (SCCL-MT1)	Cell Viability (MTT), Western Blot	IC_50_ = 80 μM	Decreased cell viability	Modulation of PTEN/AKT pathway	^21^
	Substance	Microorganism	Assay	MIC	Effect	Mechanism	Reference
Antimicrobial Activity	(+)-Medicarpin	*Trametes versicolor*	Agar dilution assay	150 mg/L	100% growth inhibition	Prevention of fungal decay	^14^
	(+)-Medicarpin	*Phytophthora megasperma*	Agar plate assay (fungal)	0.5 mM	Significant inhibition	Inhibition of fungal growth	^12^
	Medicarpin	*Helminthosporium carbonum* and *Stemphylium botryosum*	Oxygen uptake and germ tube growth assay	N/A	Inhibited oxygen uptake and germ tube growth	Inhibition of oxygen uptake and germ tube growth	^69^
	(+)-Medicarpin	*Neisseria gonorrheae*	Broth microdilution (antibacterial)	0.25 mg/mL	Selective inhibition	Selective action against *N*. *gonorrheae*	^15^
	Substance	Model	Assay	Dose/Concentration	Effect	Mechanism	Reference
Antioxidant Activity	Medicarpin	HeLa cells	ARE-luciferase activity, gene expression	50 μM	Increased antioxidant gene expression (HO-1, GCLC, NQO-1), NRF2 stabilization	Promotion of NRF2 translocation, inhibition of proteasomal degradation	^17^
Anti-Inflammatory Effects	Medicarpin	AB-CXBG mouse mastocytoma cells	Leukotriene inhibition	IC_50_ = 0.48 PM	Inhibited leukotriene C4 production	5-lipoxygenase inhibition	^16^
Metabolic Effects	Medicarpin	C3H10T1/2 mesenchymal stem cells	Browning of adipocytes	10 μM	Induced browning, upregulation of UCP1, Prdm16, Pgc-1α, enhanced mitochondrial biogenesis	Activation of AMPK pathway	^89^
		Brown adipose tissue (BAT) cells	Lipolysis in brown adipocytes	10 μM	Increased glycerol release, upregulation of HSL, ATGL, enhanced fatty acid oxidation	Activation of PKA pathway, HSL phosphorylation at serine 660	^90^

a **Abbreviation**: **ALP:** Alkaline Phosphatase. **TRAP:** Tartrate Resistant Acid Phosphatase. **NF-κB:** Nuclear Factor Kappa B.**BMP-2:** Bone Morphogenetic Protein 2. **P38MAPK:** P38 Mitogen-Activated Protein Kinase. **OPG:** Osteoprotegerin. **RANKL:** Receptor Activator of Nuclear Factor Kappa-B Ligand. **qPCR:** Quantitative Polymerase Chain Reaction. **ER:** Endoplasmic Reticulum. **PI:** Propidium Iodide. **PTH:** Parathyroid Hormone. **cAMP:** Cyclic Adenosine Monophosphate. **PKA:** Protein Kinase A. **CREB:** Cyclic AMP Response Element-Binding Protein. **RUNX2:** Runt-Related Transcription Factor 2. **BCL-2:** B-cell Lymphoma 2. **TNFα:** Tumor Necrosis Factor α. **IL-1:** Interleukin-1. **IL-6:** Interleukin-6. **OPG/RANKL:** Osteoprotegerin/Receptor Activator of Nuclear Factor Kappa-B Ligand Ratio. **Erβ:** Estrogen Receptor β. **ATF4:** Activating Transcription Factor 4. **CHOP:** C/EBP Homologous Protein. **Bax:** Bcl-2-associated X protein. **MTT:** Methylthiazolyldiphenyl-tetrazolium bromide. **BMP:** Bone Morphogenetic Protein.

Medicarpin, examined here in its isolated form, is notable for its unique properties and therapeutic potential compared with related pterocarpans and isoflavonoids, which display considerable variability in biological activity and pharmacokinetics. Evidence from several studies suggests that, despite sharing a common biosynthetic pathway, these compounds exhibit significant differences in their biological activities and pharmacokinetics. For example, maackiain and pisatin, although structurally similar, display opposite stereochemical configurations, which may influence their interactions with specific molecular targets, including quinone reductase 2 (NQO2), mitogen-activated protein kinases (MAPKs), and nuclear receptors such as ERβ Haider et al.^91–93^ Furthermore, genistein has demonstrated higher systemic bioavailability compared with daidzein, preferentially interacting with estrogen receptor β (ERβ) and modulating pathways such as PI3K/Akt and NF-κB, which may affect its therapeutic efficacy Ubaid et al.^94^ Studies on equol, a daidzein metabolite, also highlight notable differences in biological activity and pharmacokinetics among related flavonoids Setchell et al.^95^ By comparing medicarpin with these compounds, distinctive aspects of its biological activity can be identified, including potential anti-inflammatory, antioxidant, and hormonal modulatory effects, reinforcing its promise as a therapeutic alternative (Figure 6).

**Figure 6:**
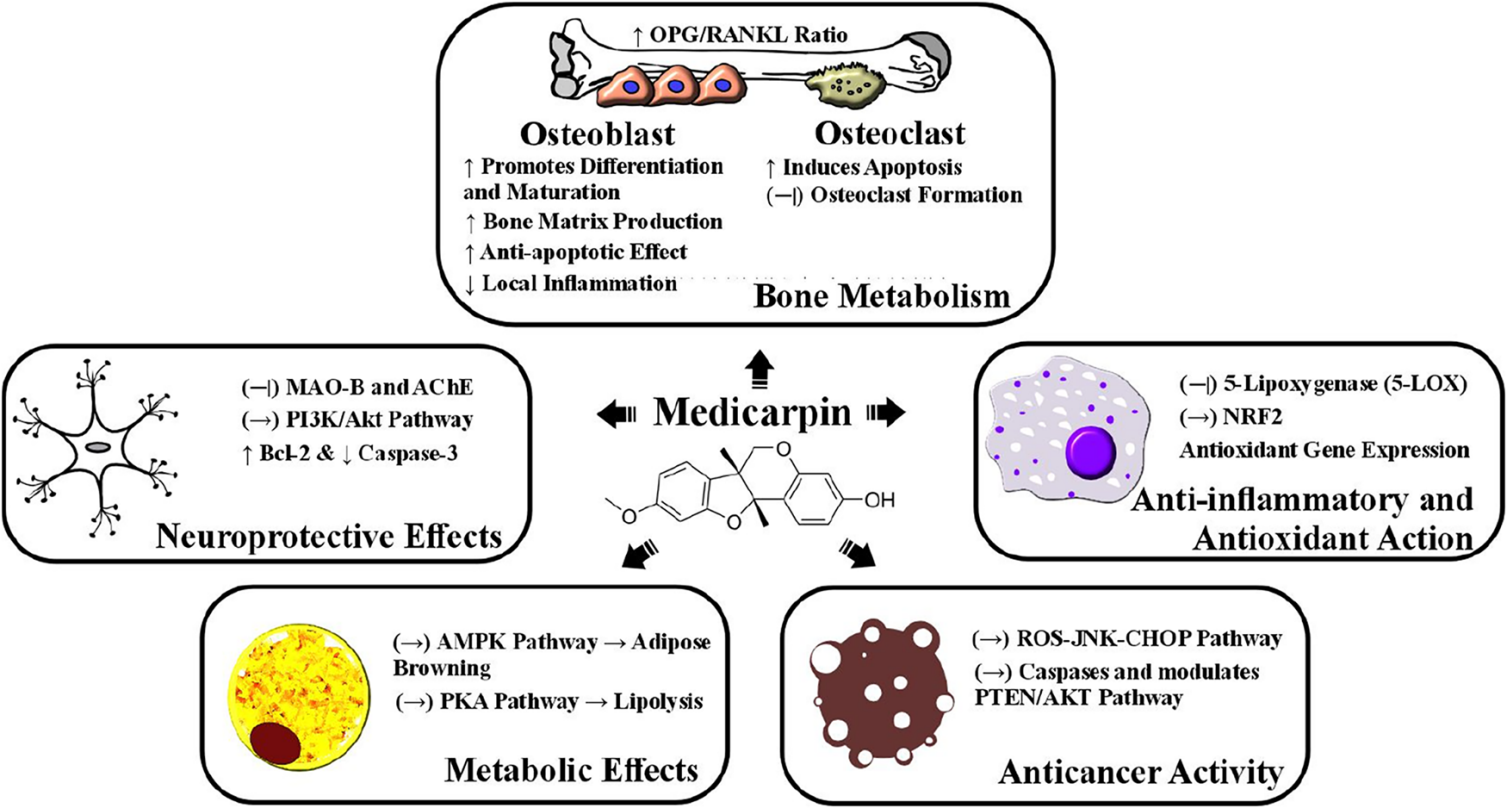
Schematic overview of the multifunctional effects of medicarpin. **Abbreviations:** AChE, acetylcholinesterase; AMPK, AMP-activated protein kinase; ERβ, estrogen receptor β; GSK-3β, glycogen synthase kinase-3β; MAO-B, monoamine oxidase-B; OPG, osteoprotegerin; PI3K/Akt, phosphatidylinositol 3-kinase/Akt pathway; PKA, protein kinase A; RANKL, receptor activator of nuclear factor kappa-B ligand. Arrows (→) indicate activation; symbols (—|) indicate inhibition.

In bone homeostasis, it promotes osteoblast-mediated bone formation and suppresses osteoclast-driven resorption. Its neuroprotective effects involve enzyme inhibition, activation of survival pathways, and antioxidant activity. Anticancer action is represented by apoptosis induction, while metabolic and anti-inflammatory roles are illustrated by pathways that increase energy expenditure and modulate inflammation (Figure 6).

## 6 Preclinical Model Assessments

As mentioned throughout the text, medicarpin is a pterocarpan found in various plants, notably those of the genus *Medicago*, such as alfalfa. Over the past few decades, this compound has garnered significant attention for its therapeutic potential, particularly in research focused on bone health, anti-inflammatory activity, and antioxidant effects (Figure 7).^30,32^

**Figure 7:**
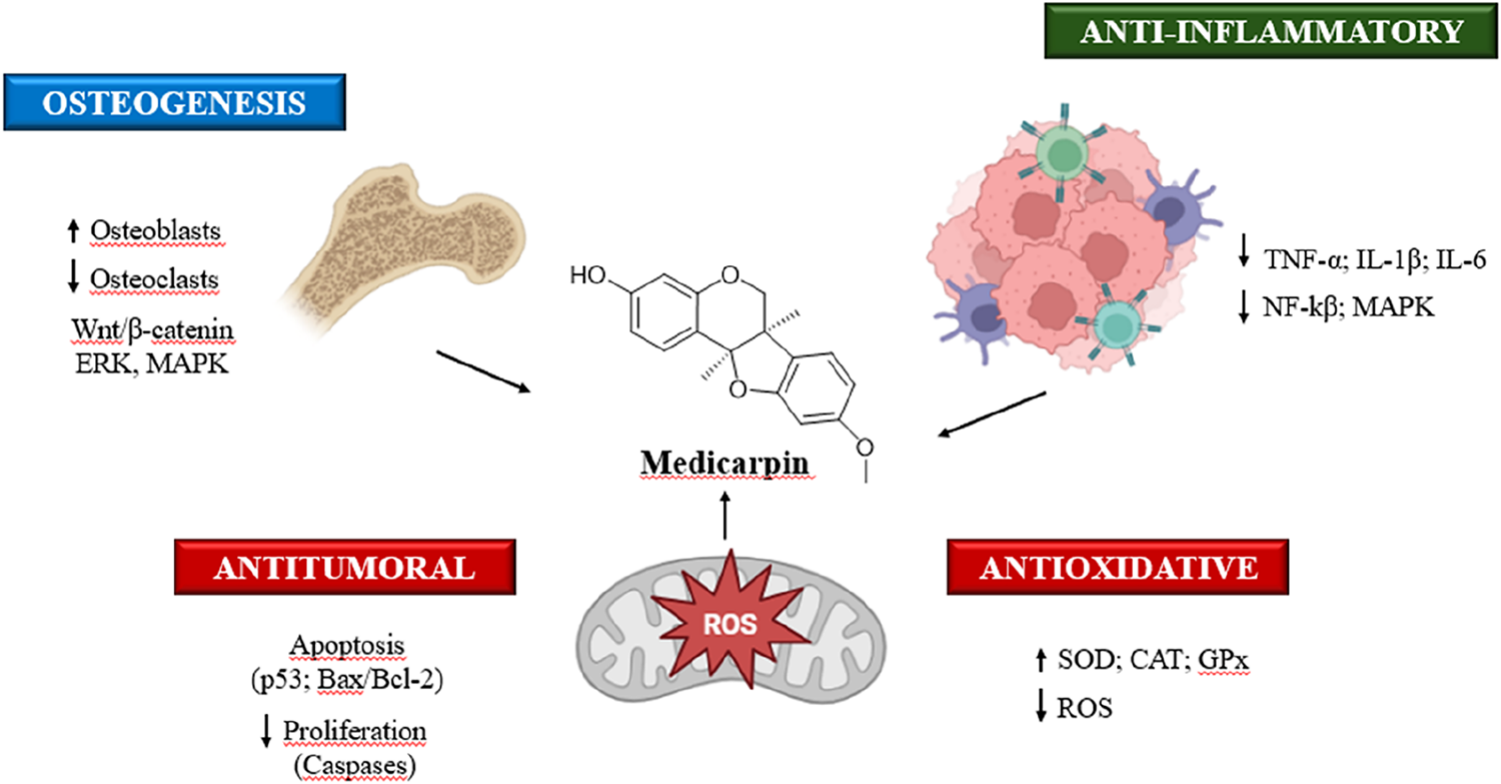
Mechanisms of medicarpin’s biological effects. Medicarpin promotes osteogenesis, exerts anti-inflammatory and antioxidant actions, and induces apoptosis in tumor cells through distinct molecular pathways. *Created with BioRender*.*com*.

Regarding preclinical model assays with this compound, there are several interesting points to highlight. Studies have focused on its osteogenic properties, anti-inflammatory effects, and antioxidant effects. Additionally, as observed with other isoflavones, this compound has shown significant phytoestrogenic activity.^30,32,34,36^ Moreover, animal assays have been used to evaluate the toxicity and potential adverse effects of higher doses and concentrations of Brazilian red propolis, with medicarpin as one of its major componentes.^96^ These studies are crucial, especially when used as a basis to determine the safety range before clinical trials in humans. However, to date, no studies have been reported on preclinical toxicological trials involving isolated medicarpin.

Tyagi and collaborators^34^ evaluated the effect of medicarpin on adult female Balb/c mice (9–10 weeks old) over a period of 6 weeks. They observed thymic atrophy and a reduction in the ovariectomy-induced increase in serum levels of TNF-α and its mRNA levels in T cells from bone marrow. Medicarpin/Estrogen reduced the proliferation of CD4+ T cells in the bone marrow and spleen, preventing the loss of CD28 receptors on CD4+ T cells. Furthermore, medicarpin abolished the loss of CD28 expression induced by TNF-α in T cells from the bone marrow. The study demonstrated that ovariectomy leads to the generation of premature senescent CD4 + CD28null T cells, an effect inhibited by estrogen and medicarpin. The authors suggested that one of the mechanisms by which medicarpin/estrogen alleviates ovariectomy-induced bone loss is by delaying T cell senescence and increasing CD28 expression.

In recent years, there has been growing interest in the potential benefits of this bioactive pterocarpan. However, its metabolic fate in vivo remains largely unknown. To address this, Wang and collaborators conducted a study in 2019 to clarify its metabolism and the distribution of its metabolites in a preclinical model. They identified approximately 165 new metabolites, including 13 phase I metabolites and 152 phase II metabolites, following oral administration, using HPLC-ESI-IT-TOF-MSn.^37^

The study was conducted with 8 male Sprague–Dawley rats (220–250 g), randomly divided into two groups before compound administration by orogastric gavage: a treatment group and a control group, with four rats per group. Each rat was housed in a metabolic cage with water and food ad libitum for 3 days. Medicarpin was suspended in a 0.5% CMC-Na solution and administered at a dose of 100 mg/kg (body weight) to the treatment group once daily for 4 days, while the control group received an equivalent volume of 0.5% CMC-Na. The metabolites were detected in various matrices, including urine, feces, plasma, colon, intestine, stomach, liver, spleen, kidneys, lungs, heart, brain, and thymus. The metabolic reactions observed included demethylation, hydrogenation, hydroxylation, glucuronidation, sulfation, methylation, glycosylation, and conjugation with vitamin C. Two metabolites, M1 (medicarpin glucuronide) and M5 (vestitol-1′-O-glucuronide), were found in 10 organs, with M1 being the most abundant in seven of them. According to the authors, based on information available in the SciFinder database, approximately 93 metabolites were identified as potentially new compounds.^37^

In 2020, Taneja and collaborators investigated the oral pharmacokinetics, tissue distribution, and excretion of medicarpin after the administration of a single oral dose in female rats (Sprague–Dawley, weighing 220 ± 20 g). The oral pharmacokinetics were explored at doses of 5 and 20 mg/kg, while tissue distribution, urinary excretion, and fecal excretion were studied after the oral dose of 20 mg/kg. Medicarpin was quantified in plasma, urine, feces, and tissue samples from the rats using a validated LC-MS/MS method, after separation by reverse-phase HPLC on an RP18 column (4.6 mm × 50 mm, 5.0 μm). They found a low oral bioavailability of medicarpin, with systemic levels also considered low. However, the concentration in tissues was significantly higher than that found in plasma, with the highest concentrations in the liver, followed by the bone marrow. The urinary and fecal excretion of medicarpin was less than 1%, indicating high distribution in body tissues and minimal excretion via urine or feces. The authors suggested that the low excretion in both urine and feces implies metabolism is the main route of elimination, with first-pass metabolism in the intestine likely causing the low oral bioavailability.^36^

The gastroprotective effect and mode of action of both the hydroalcoholic extract of red propolis and its main compounds, including medicarpin, have also been evaluated. Boeing and colleagues^97^ assessed the effect of this extract—30, 100, and 300 mg/kg orally and 30 mg/kg intraperitoneally—and its isolated compound medicarpin—10 mg/kg orally—in male Swiss mice (25–30 g, aged between 3 and 4 months) and male Wistar rats (200–250 g, aged between 3 and 4 months). The authors found that the red propolis extract exhibited significant gastroprotective activity in mice induced with gastric ulcers, an effect mediated by prostaglandins and mucin production. According to the authors, the effect obtained from the extract may be related to the combined effects of its isolated compounds, such as methylvestitol and medicarpin. These compounds exhibited synergistic effects in some evaluated parameters and complementary effects in others, thus reinforcing the use of propolis in treating gastric disorders. Additionally, medicarpin itself displayed a significant gastroprotective effect evidenced by the reduction of lesions.

In 2021, Aldana-Mejía and coauthors, while evaluating the possible toxic effects of a Brazilian red propolis sample—with medicarpin as one of its major compounds—on zebrafish, observed altered behavior in animals treated with extract concentrations ranging from 12.5 to 100 mg/L. Lack of balance, accelerated operculum movements, and altered buoyancy were some of the changes leading to mortality. However, at lower concentrations (0.8 to 6.3 μg/mL), no behavioral or physiological changes were noted. The calculated LC_50_ was 9.37 μg/mL.^96^

Table 4 provides a summary of the main studies conducted to date on the effects and potential activities associated with medicarpin.

**Table 4:** Biological Activities Evaluated *In vivo*Regarding The Effects Of Medicarpin And Its Derivatives^a^

	study	animal model	methods	experimental model and dose	comments	references
*In vivo*	Bone metabolism	Female BALB/c mice (9–10 weeks old)	qPCR, Flow cytometry, ROS measurement (DCF-DA - 10 μg/mL concentration)	Vehicle (gum acacia in distilled water); Ovx + vehicle; Ovx +10. 0 mg kg^–1^ day^–1^ Med; and Ovx +0.01 mg kg–1 day–1 E2. All treatments were given by oral gavage/6 weeks.	Medicarpin and estrogen improve bone loss in ovariectomized animals by delaying T cell senescence and increasing CD28 expression.	^34^
	Toxicological	Male and female adult zebrafish (*Danio rerio*) (6 months old/0.35 ± 0.18 g, body size 3.12 ± 0.70 cm)	Acute Toxicity Assay (OECD 203 recommendations)	0.8, 1.6, 3.1, 6.3, 12.5, 25, 50, and 100 mg/L, for 96 h in a static system.	Concentrations of 0.8–6.3 mg/L of Brazilian red propolis were safe for the animals, with a LC_50_ of 9.37 mg/L.	^96^
	Metabolism and tissue distributions	Male *Sprague–Dawley* rats (220–250 g)	Urine and feces samples were collected for the first 3 days after drug administration; plasma samples and organ samples were collected on the last day 1 h after drug administration/HPLC-ESI-IT-TOF-MSn analysis	Medicarpin (0.5% CMC-Na solution)/100 mg/kg (body weight) to the drug group rats/1x/day/4d; blank group: same volume of 0.5% CMC-Na.	The metabolic reactions of medicarpin included demethylation, hydrogenation, hydroxylation, glucuronidation, sulfation, glycosylation, methylation and conjunction of vitamin C, among which sulfation and glucuronidation were the major phase II metabolic reactions.	^37^
	Bioavailability, tissue distribution and excretion	Female *Sprague–Dawley* rats, (220 ± 20 g).	Tissue distribution and excretion study; *In vitro* urine and feces stability	5 and 20 mg/kg. At 0.08, 0.25, 0.5, 0.75, 1, 2, 3,5, 7, 9, and 24 h postdosing	The low amounts of medicarpin excreted in urine and feces suggest that metabolism is the main route of elimination of this compound and the first-pass metabolism in the intestine could be a probable reason for the low oral bioavailability of medicarpin.	^36^
	Gastroprotective property against ethanol/HCl-induced damage	Male Swiss mice (25–30 g, aged/3 and 4 months) and male Wistar rats (200–250 g, aged/3 and 4 months)	Chromatographic analysis and isolation of compounds; Determination of gastric secretion; Evaluation of the gastroprotective mechanisms; Histological analysis; Determination of oxidative and inflammatory parameters	Naive (received no treatment); Vehicle (negative control, water plus 1% tween, 10 mL/kg, p.o.); Carbenoxolone (positive control, 200 mg/kg, p.o.), HERP at three different dosages (30, 100, and 300 mg/kg, p.o. or 30 mg/kg, i.p.)	Effect is dependent on prostaglandins and mucin production. The compounds methylvestitol, medicarpin may have an essential role in the activity of extract.	^97^
	Neuroprotective effect	Male Kunming mice (7–8 weeks weight, 25–30 g)	The Morris water maze test; New object recognition test; Biochemical analysis; Western blotting (···)	Control group; Scopolamine group; Scopolamine + low-dose Medicarpin group, treated with Medicarpin at 2.5 mg/kg; (4) Scopolamine + high-dose Medicarpin group, treated with Medicarpin at 5 mg/kgmg/kg28 days; Scopolamine + Donepezil group (Don), Donepezil at 3. Oral gavage for	Network analysis and Western blotting further identified two key targets, GSK-3β and MAPK14 (p38), in the AD-related protein regulatory network, which play key roles in the regulation of neuronal apoptosis and synaptic plasticity by Medicarpin	^88^
	Antitumor effect	Male/Female BALB/c nude mice (4–6-week-old, 20 g ± 2 g body weight)	CCK-8 Analysis; Colony Formation Analysis; Transwell Assay; Real-Time PCR; Western blotting.	Model group: intragastric administration of normal saline Once a day, for 7 days; Low-dose JPFR group: JPFR decoction gavage (once a day, a total of 28 days); (3) Medium dose JPFR group: JPFR decoction gavage (once a day, a total of 28 days); (4) High-dose JPFR group: JPFR decoction gavage (once a day, a total of 28 days); and (5) a Positive control group (L–OHP): intraperitoneal injection of oxaliplatin 0.6 mg/kg mL (once every other day, for 28 days).	It has been demonstrated that JPFR contains many effective compounds, including medicarpin, that can directly target cancer-associated signaling pathways. Both *in vitro* and *in vivo* assays further confirmed that JPFR can inhibit the growth and metastasis of LoVo colorectal cancer cells by regulating genes or proteins associated with β-catenin signaling.	^98^
	Bone metabolism	Female *Sprague–Dawley* rats (21 days old) (200–220 g)	Assessment of various bone parameters and uterine estrogenicity; Plasma pharmacokinetics.	Medicarpin was administered a 5.0 mg·kg^–1^ bolus dose by oral gavage. Blood samples were collected from the retro-orbital plexus of rats into microfuge tubes containing heparin as an anticoagulant at 0.08, 0.25, 0.50, 1, 2, 4, 6, 8, and 10 h postdosing.	Medicarpin stimulates osteoblast differentiation, via ERβ, promotes the attainment of peak bone mass, and is free from uterine estrogenicity. Furthermore, due to its excellent oral bioavailability, Med holds promise as a potential osteogenic agent.	^28^
	Immunomodulatory effect	Female DBA/1J (8–12 weeks old) mice	Histological studies; CBA cytokine assay and COMP Elisa Assay	DBA/1J mice were immunized with 100 μL of type-2 collagen (200 μg) with FCA on day 0 and booster dose with IFA on day 21. After day 21 treatment of Medicarpin (10 mg/kg/body weight) and anti-TNF-α (1 mg/mice) was given for one month.	Medicarpin was effective in preventing postmenopausal polyarthritis by modulating TH-17 and Treg cells, thereby preventing cartilage destruction and bone erosion.	^84^
	Bone metabolism/Immunomodulatory effect	Female BALB/c mouse.	Immunoblotting studies.	Sham operated (ovary intact); vehicle (gum acacia suspended in distilled water); Ovx: Vehicle; Ovx: 10.0 mg/kg/day medicarpin. Daily oral treatment/1 month.	Medicarpin inhibits ER stress-induced apoptosis and promotes osteoblast cell survival by targeting GRP78.	^87^
*Ex vivo*		Rat Preosteoblasts	Co-immunofluorescence, qPCR, Western blotting	Not reported	Activation of Notch and Wnt canonical signaling pathways, upregulation of β-catenin and Notch-1, and downregulation of GSK-3β, colocalization of β-catenin with alkaline phosphatase.	^29^
		Mouse bone marrow cells	Osteoclastogenesis assay, TRAP staining	10^–10^ M to 10^–8^ M medicarpin	Estrogen receptor-independent mechanism, reduction in TNFα and NF-κB signaling	^33^
		Bone marrow cells (1 × 10^6^/mL) from C57BL/6 mice (6–8 weeks old)	Immunoblotting studies/qPCR	BMMC (5 × 10^6^) were pretreated With DMSO (0.1%) or the 8 kinds of flavonoids (30 μM) for 1 h at 37 °C prior to the stimulation with IL-33 (10 ng/mL) for 30 min. Nuclear extracts were prepared and immunoblotted with an anti-NF-κB p65 antibody or anti-Lamin B antibody as a control.	(+)-Medicarpin, failed to inhibit the IL-33-induced activation of NF-κB	^99^

a **Abbreviation**: **OECD:** Organization for Economic Cooperation and Development Guidelines for the Testing of Chemicals. **CD:** Cluster of Differentiation. **LC50:** Lethal Concentration 50. **CMC-Na:** Sodium Carboxymethylcellulose. **HERP:** extract of red propolis. **GSK-3β:** Glycogen Synthase Kinase 3 β. **p38 MAPK14**: Mitogen-Activated Protein Kinase 14. **RT-PCR:** Reverse Transcription Polymerase Chain Reaction. **Ovx:** Ovariectomy. **CCK-8:** Cell Counting Kit-8. **IL:** Interleukin. **ROS:** reactive oxygen species. **JPFR:** JianPi Fu Recipe. **BMMC:** bone marrow-derived mast cells. **DMSO:** dimethyl sulfoxide.

In summary, these in vitro and in vivo assays underscore the therapeutic potential of this compound and emphasize the need for new trials to thoroughly evaluate its benefits in human clinical trials. Furthermore, these studies aim to identify effective methods for administering medicarpin, thereby allowing it to achieve its full biological potential.

## 7 Discussion and Expectation

As mentioned throughout the text, various studies have investigated medicarpin in different areas. However, the literature still lacks comprehensive data on certain aspects, particularly concerning pharmacokinetic, toxicological, and clinical evaluations. Despite its promising pharmacological properties, detailed information on the toxicological potential of medicarpin remains scarce. Most studies have addressed this issue indirectly, focusing on its selectivity—specifically, its ability to target cancer cells while sparing healthy cells. However, these findings are insufficient to definitively establish its safety profile.

In the search for novel agents capable of enhancing the effects of existing anticancer treatments, medicarpin was studied alone and in combination with tumor necrosis factor-related apoptosis-inducing ligand (TRAIL) against primary myeloid leukemia cells and primary human peripheral blood mononuclear cells (PBMCs). Cell viability and cell death assays (LDH release) of PBMCs showed no significant differences when treated with medicarpin (20 μM) and TRAIL (2.5 ng/mL), either individually or in combination, compared to the control group.^20^

Similarly, selective effects were observed in studies evaluating medicarpin’s cytotoxicity against bladder cancer cells in vitro. Medicarpin, in concentrations up to 200 μM did not show cytotoxicity toward SV-HUC-1, a normal immortalized human bladder epithelial cell line. These results suggest that medicarpin effectively inhibits the proliferation of bladder cancer cells while exhibiting minimal toxicity against normal bladder epithelial cells.^18^ Additionally, medicarpin was not toxic to normal Madin-Darby Canine Kidney (MDCK) cells at concentrations up to 50 μM, but exhibited selective toxicity against neuroblastoma cells (SH-SY5Y).^25^

While these studies provide valuable indirect evidence of medicarpin’s selectivity, they do not offer comprehensive toxicological evaluations. As Sharma and collaborators^31^ pointed out, significant gaps remain between preclinical, clinical, and regulatory studies. Although experimental animal models were promise, with suggested doses of 24 mg daily for a 60 kg individual, these findings cannot be translated into clinical practice without rigorous safety and toxicity assessments.^31^

Similarly, pharmacokinetic studies have underscored the importance of repeat-dose toxicological studies to evaluate potential adverse effects. This suggests that its metabolism—primarily occurring in the liver and gut through oxidation, demethylation, and glucuronidation, as mentioned throughout the text—is responsible for its low oral exposure.^36^ However, its accumulation in the liver, bone marrow, and intestine, combined with the lack of toxicological data, raises concerns about the compound’s true effects.

Despite its promising pharmacological potential, the development of medicarpin as a therapeutic agent is currently limited by the lack of robust toxicological and pharmacokinetic (PK) studies. Such evaluations are critical for defining safety margins, adverse effects, and exposure profiles, all of which are prerequisites for clinical trials and regulatory approval. Without them, the therapeutic promise of medicarpin remains speculative.

Rodent studies provide the most detailed insights to date. Medicarpin is rapidly absorbed following oral dosing, with a short Tmax (∼15 min), low oral bioavailability (∼17% in rats), and multipeak plasma profiles suggestive of complex disposition processes. High-resolution MS^n^ metabolite mapping has identified over 150 metabolites, with glucuronidation and sulfation as the dominant clearance routes. Circulating and tissue-associated signals consist primarily of conjugated metabolites, not the parent compound, indicating that in vivo effects may be mediated largely by this metabolite pool.^35–37^ Available ADME data further show broad tissue distribution, high plasma protein binding, and dual renal and biliary elimination. Sulfate conjugates dominate urinary excretion, while glucuronides prevail in plasma; unusual conjugates and isomerization products have also been reported, underscoring the metabolic complexity of medicarpin.^100^

Despite these advances, critical gaps remain. Standard PK parameters (AUC, Cmax, clearance, absolute bioavailability) are incompletely defined outside of rodents, and no human ADME data exist. The pharmacology and toxicology of major conjugates remain unknown, leaving it uncertain whether efficacy and safety depend on the parent compound or metabolites. Moreover, enzyme and transporter phenotyping (UGTs, SULTs, CYPs, P-gp, BCRP, OATs/OATPs) has not been performed, limiting the prediction of drug–drug interactions or strategies to enhance systemic exposure.

To enable translation, a focused research strategies is required: (i) in vitro enzyme and transporter phenotyping; (ii) synthesis and pharmacological evaluation of key metabolites; (iii) mechanistic rodent studies on enterohepatic recycling and microbiome contributions; (iv) nonrodent PK/ADME studies; and (v) first-in-human PK investigations using validated LC–MS/MS assays. These steps will provide the exposure, clearance, and metabolite data essential to support clinical development.^101,102^

In addition to the need for such approaches to be carried out in the contexts mentioned above, there is a growing demand for bioactive compounds to be better assimilated by the body. To this end, the increasing use of certain technologies, particularly nanotechnology, is necessary.^103^ Recent studies and reviews reported that this technology can enhance the bioavailability and biodisponibility of bioactive compounds.^50,104–108^ Comparative studies with clinically approved compounds could further help anticipate translational potential by providing benchmarks for efficacy and safety, thereby bridging the gap between preclinical studies and clinical application.^109^

Although several nanotechnology-based formulations have been reported for flavonoids and Brazilian red propolis—including polymeric nanoparticles,^110–112^ nanoemulsions,^113^ gold nanoparticles,^114^ mesoporous silica nanoparticles,^115^ biocompatible polymeric matrices,^110^ and polymeric films^116^—specific formulations of medicarpin remain scarce. Nevertheless, patents and intellectual property (IP) highlight its translational potential. A Brazilian filing (BR102023017466A2)^117^ describes nanobeads loaded with red propolis extract for dermocosmetic and therapeutic uses, supporting the concept of “nanopropolis.” By contrast, US patent 10,385,061 ^118^ claims medicarpin itself, its synthesis, and applications. This is a strategic distinction: while red propolis patents focus on extracts and formulations, medicarpin is positioned as a standalone, high-value pharmaceutical candidate. Such patents illustrate how natural molecules, though unpatentable in raw form, can be protected through novel uses or synthetic derivatives, strengthening their value in drug development programs.

Despite this progress, there is still a lack of literature on applying nanotechnology directly to medicarpin. Promising opportunities include optimizing nanostructures, enhancing dosing efficiency, and developing controlled-release implants or thin films within nanostructured matrices, particularly relevant given medicarpin’s osteogenic potential. Lipid- and polymer-based nanoparticles could improve bioavailability, targeted delivery, and stability, while controlled-release systems may reduce dosing frequency and enhance patient compliance.^119,120^

Functional foods and nutraceuticals represent another feasible route for human administration, with nanotechnology improving solubility and effectiveness to support daily health regimens.^121,122^ Equally important is the dietary relevance of medicarpin and related isoflavonoids naturally found in legumes such as soy, chickpea, and alfalfa sprouts, as well as Brazilian red propolis.^123–125^ These foods are widely consumed, contribute to bone health, and may aid in the prevention of osteoporosis.^126^ Although epidemiological evidence directly addressing medicarpin is limited, legumes are established functional foods, supporting the hypothesis that dietary intake contributes to health promotion. Future research should assess the role of dietary medicarpin, investigate nanotechnological delivery strategies, and define its place within the broader context of legume-derived bioactives in both pharmacological and preventive nutrition.

## Supplementary Material



## Notes

**Table A**. Summary of extraction and isolation methods for medicarpin (Extraction technique, Part, Solvet, Raw material, Isolation/purification, Yield, Pros and Cons); **Table B**. Summary of analytical methods for identification and determination of medicarpin (Method, Analyzed sample, Purpose, Equipment and conditions, Pros and Cons) (PDF)

## Abbreviations

2HF: 2′-hydroxyformononetin
2MF: 2′-methoxyformononetin
4CL: 4-coumarate:CoA ligase
AChE: acetylcholinesterase
**AlCl**_**3**_: aluminum chloride
ALP: alkaline phosphatase
AMPK: AMP-activated protein kinase
ARE: antioxidant response element
ATF4: activating transcription factor 4
ATGL: adipose triglyceride lipase
BAT: brown adipose tissue
Bax: Bcl-2 homologous antagonist/killer
BCL-2: B-cell lymphoma 2
BDNF: brain-derived neurotrophic factor
**BF**_**3**_ **-OEt**_**2**_: boron trifluoride-ether
BMMC: bone marrow-derived mast cells.
BMP: bone morphogenetic protein
BMP-2: bone morphogenetic protein 2
C4H: cinnamic acid 4-hydroxylase
cAMP: cyclic adenosine monophosphate
CCK-8: cell counting kit-8
CD: cluster of differentiation
**CH**_**3**_ **CN**: acetonitrile
CHI: chalcone isomerase
CHI II: chalcone isomerase II
CHOP: C/EBP homologous protein
CHR: chalcone reductase
CHS: chalcone synthase
CMC-Na: sodium carboxymethylcellulose
Col1a1: collagen type i α 1 chain
CREB: cyclic amp response element-binding protein
CUMS: chronic unpredictable mild stress
DMF: dimethylformamide
DMI: 7,2′-dihydroxy-4′-methoxy- isoflavanol
DMID: 4′-methoxyisoflavanol dehydratase
DMSO: dimethyl sulfoxide
ER: endoplasmic reticulum
ERβ: estrogen receptor β
Fluo-3-AM: fluorescent dye used to measure intracellular calcium
FNSII: flavanone synthase II
GRP78: glucose-regulated protein 78
GSK-3β: glycogen synthase kinase-3 β
HERP: extract of red propolis
HI4′OMT: isoflavone 4′-O-methyltransferase
HNSCC: head and neck squamous cell carcinoma
HPLC: high-performance liquid chromatography
HSL: hormone-sensitive lipase
I2′H: isoflavone 2′-hydroxylase
**IC**_50_: inhibitory concentrations
IFR: isoflavone reductase
IFS: isoflavone synthase
IL: interleukin
IR: infrared spectroscopy
JPFR: Jianpi fu recipe.
LC50: lethal concentration 50
LEF-1: lymphoid enhancer-binding factor 1
MAO-B: monoamine oxidase-B
**MeSO**_**2**_: methanesulfonyl chloride
MPLC: medium-pressure liquid chromatography
MS: mass spectrometry
MTT: methylthiazolyldiphenyl-tetrazolium bromide
**NaBH**_**4**_: sodium borohydride
NF-κB: nuclear factor-kappa-β
NMR: nuclear magnetic resonance
NO: nitric oxide
OECD: organization for economic cooperation and development guidelines for the testing of chemicals
OPG: osteoprotegerin
OPG/RANKL: osteoprotegerin/receptor activator of nuclear factor kappa-β ligand ratio
ORD: optical rotatory dispersion
Ovx: ovariectomy
p38-MAPK: p38 mitogen-activated protein kinase
PAL: phenylalanine ammonia lyase
Pb: lead
PCMBA: *p*-chloromercuribenzoic acid
p-CREB: phosphorylated CREB
PI: propidium iodide
PKA: protein kinase a
PTH: parathyroid hormone
qPCR: quantitative polymerase chain reaction
RANKL: receptor activator of nuclear factor kappa-β ligand
ROS: reactive oxygen species
RP: reverse phase
RT-PCR: reverse transcription polymerase chain reaction
RUNX2: runt-related transcription factor 2
SA: salicilic acid
TLC: thin layer chromatography
TNF-α: tumor necrosis fator- α
TRAP: tartrate resistant acid phosphatase
UCP1: uncoupling protein 1
UV: ultraviolet spectroscopy
VR: vestitone reductase
