# Supplementary material for: A Comprehensive Review of Medicarpin: A Phytoalexin with Therapeutic Potential
Source: ACS Omega. 2025 Nov 7;10(45):53722–45. doi: 10.1021/acsomega.5c08170 (PMC12631411; doi:10.1021/acsomega.5c08170)
Supplement: Supplementary file 1 [file ao5c08170_si_001.pdf]

A Comprehensive Review of Medicarpin: A Phytoalexin with Therapeutic Potential

Matheus Hikaru Tanimoto[1], Aline Mayrink de Miranda[2], Jennyfer Andrea Aldana-Mejía[3], Luciana Silva de Araújo[1], Ana Maria de Freitas Pinheiro[1], Ana Fernanda Guimarães Trindade[1], Júlia Mina Fernandes[1], Samir A. Ross[3,4], Jairo Kenupp Bastos[1*]

[1] School of Pharmaceutical Sciences of Ribeirão Preto, University of São Paulo, Ribeirão Preto, SP, 14040-903, Brazil.
[2] Faculty of Pharmaceutical Sciences, University of São Paulo, São Paulo, SP, 05508-000, Brazil.
[3] National Center for Natural Products Research, School of Pharmacy, The University of Mississippi, Oxford, MS 38677, USA.
[4] Department of Biomolecular Sciences, Division of Pharmacognosy, School of Pharmacy, University of Mississippi, Oxford, MS 38677, USA.

***Corresponding author**
Tel.: +55-16-3315-4879.
E-mail address: jkbastos@fcfrp.usp.br

Table A. Summary of extraction and isolation methods for medicarpin.

Extraction technique	Part	Solvent (extraction time)	Raw material-to-solvent-ratio	Isolation / purification	Yield	Reference	Pros	Cons
Maceration	Roots	Methanol	12 kg roots/60 L. 66 g of neutral fraction.	Charcoal column chromatography with acetone → crystals washed with ethyl acetate, methanol and benzene → Hydrolysis of medicarpin-β-D-glucoside → partition with ethyl acetate → wash with acetone → silica gel TLC for isolation	1.48 g of medicarpin-β-D-glucoside/66g extract.<br><br>5.3 mg of medicarpin/100mg of medicarpin-β-D-glucoside	1	Simple method, no need for complex equipment.<br><br>Does not require high technical skills.<br><br>Low energy consumption.<br><br>Long contact time allows extraction of poorly soluble compounds.<br><br>Suitable for low-cost and less potent extracts.<br><br>Preserves thermolabile compounds by avoiding high temperatures.<br><br>Maintains a chemical profile close to the raw material.<br><br>[2,3]	The process may extend over several days/weeks.<br><br>Complete recovery of compounds is often not achieved.<br><br>Slow and time-consuming compared to other techniques.<br><br>Requires relatively large volumes of solvent.<br><br>Risk of microbial growth during long extraction periods.<br><br>Possible degradation of sensitive compounds due to prolonged exposure.<br><br>Lower efficiency compared to modern extraction methods.<br><br>[3,4]
	Infected tissues	95% methanol	0.1-0.5g /20 mL	Purification through Silica gel TLC	NR	5		
	Uninoculated leaves	75% ethanol	5g / 125 mL	Partition with carbon tetrachloride → partition with ethyl acetate → purification through silica gel TLC	NR	5		
	Leaf tissue	95% ethanol	0.8-2g / 20 mL	Partition with carbon tetrachloride → partition with NaOH → acidification → partition with carbon tetrachloride → isolation through TLC	NR	6		
	Fungus infected seedlings	95% of ethanol	10-20 g/10 mL	Partition with chloroform → chloroform fraction partitioned with NaOH → acidification with HCl → partition with chloroform → purification through TLC	NR	7		
	Seedlings	95% ethanol	2 mL / g plant tissue	Partition with ethyl acetate → TLC analysis	NR	8		
	Infected plants	95% ethanol and ethyl acetate	NR	TLC separation	NR	8		

	Fungus infected leaflets	60% methanol	15 mL/g fresh tissue	Isolation with semi-preparative HPLC	15 - 110 µg/g fresh tissue	9		
	Foliage	95% ethanol	NR	Silica gel column → preparative HPLC with RP column	NR	10		
	Infected leaves	60% aqueous methanol	15 mL/g	Partition with ethyl acetate → purification with preparative HPLC	NR	11		
	Seedlings	80% methanol	1:10 w/v	Purification through semi-preparative HPLC	NR	12		
	Roots	80% methanol	1:10 w/v	NR	NR	13		
	Roots	Ethanol	4.8 Kg/28 L	Partition with ethyl acetate → MPLC on a C18 gel column → Enriched fraction submitted to a silica gel column → HPLC on a C18 column	297.5 mg	14		
	Plant pods	Methanol	1.2 Kg / 20L	Partition with Ethyl acetate → MPLC with RP open column → preparative HPLC with C18 column	22.5 mg	15		
	Cell suspension cultures	Cold acetone (-20 ℃), followed by methanol/acetone (1:1)	600 g / NR	Polyamide chromatography → preparative HPLC with RP column	NR	16		
	Plant treated with lead	80% methanol	1:10 (w/v)	Centrifugation and collect of supernatant with medicarpin	NR	17		
	Leaf tissue	Liquid nitrogen followed by 80% methanol	100g / mL	Treatment with 25 U β-glucosidase in citrate-phosphate buffer (pH 5.2) at 37°C for 10–12 h in the dark	NR	18		
	Jamaican multifloral propolis	Sequentially extracted with hexane, ethyl	750 g/2 L	Ethyl acetate portion submitted to flash chromatography → purification of a fraction by	42 mg	19		

		acetate, methanol		NP chromatography and isolation of (+)-medicarpin				
NR	Diffusates (inoculum drops)	Tetrachloride (CCl4)	NR	Partition with carbon tetrachloride → silica gel TLC	NR	5		
	Leaf diffusate	CCL4 and ethyl acetate	NR	The fraction was submitted to silica gel TLC for isolation	NR	6		
	Fungus infected seedlings	NR	NR	Purification with Sephadex	NR	20		
	Protoplast culture medium	Cold acetone	NR	Extraction with ethyl acetate	NR	21		
	Roots	NR	NR	Purification through semi-preparative HPLC with a C18 column		11		
	Roots	Ethyl acetate	NR	Purification through semi-preparative HPLC	NR	13		
	Cell suspensions	Ethyl acetate	NR	Purification through semi-preparative HPLC	NR	12		
	NR	NR	NR	Silica gel column → reversed phase C19 silica gel column	NR	22		
	Fungus infected tissue	Diethyl ether	2.5 kg/NR	Silica gel PLC for isolation	7 mg	23		
	Inoculation fluid with fungus infected seedlings	95% ethanol	2 mL ethanol/g of plant tissue	Partition with ethyl acetate → silica gel TLC → TLC bioassay	1.64 - 9.61 ug/g fresh wt.	24		
	Inoculation fluid with fungus infected leaflets	Ethyl acetate	240 mL inoculation fluid/240 mL ethyl acetate	Partition with TLC	NR	25		

	Fungus infected leaves	Chloroform	NR	Partition of the drop diffusates with silica gel TLC	NR	26		
	Inoculation fluid with fungus infected leaves	Chloroform-Sodium hydroxide	NR	TLC → LH-20 Sephadex chromatography column	NR	26		
	Fungus infected leaflets / treated leaflets with Copper (II) sulfate	Ethyl acetate	NR	Isolated with silica gel TLC	50 µg/g fresh tissue from fungus infected leaflets and 80 ug/g fresh tissue from treated leaflets with CuSO4	9		
	Plant extract	Chloroform	NR	Sephadex LH-20 chromatography → LTC4 inhibition in the CXBG assay → Preparative HPLC	NR	27		
Soxhlet extraction	Heartwood	Successive extraction with hexane (6h each)	20g	Spontaneous crystallization of medicarpin → Dichloromethane washing → Crystals heated at 55 ℃ for 12 h.	NR	28	Simple and efficient technique.<br><br>Allows extraction of large amounts of plant material.<br><br>No need for filtration after extraction.<br><br>Suitable for both small-scale and large-scale applications.<br><br>Requires less solvent than maceration or similar methods.<br><br>Ensures continuous contact between “fresh” solvent and material, which improves extraction efficiency.<br><br>Enables exhaustive extraction of compounds with limited	Not suitable for highly volatile compounds.<br><br>Risk of thermal degradation of heat-sensitive compounds.<br><br>Extraction process can be lengthy.<br><br>Labor-intensive procedure.<br><br>Limited flexibility to modify extraction parameters once the system is running.<br><br>Requires relatively large volumes of solvent compared to modern techniques.

							solubility.<br><br>Operates under controlled temperature, reducing the risk of degradation compared to open boiling.<br><br>[2,4,29]	Continuous heating may increase energy consumption.<br><br>Glassware setup is fragile and requires careful handling.<br><br>[2,29,30]
Ultrasound-assisted extraction	Roots	70% methanol	4 g/400 mL	Centrifugation, supernatant collected and filtrated with medicarpin	NR	[31]	Higher efficiency compared with conventional methods.<br><br>Ensures uniform mixing and minimizes thermal gradients.<br><br>Requires smaller volumes of solvent.<br><br>Consumes less energy and solvent overall.<br><br>Improves extraction yield.<br><br>Short process operates at lower temperatures, preserving thermolabile compounds.<br><br>Suitable for both small-scale laboratory work and larger-scale applications.<br><br>[2–4,30]	Heat generated during long process may degrade thermolabile compounds.<br><br>Efficiency depends on the position of the sample container within the ultrasonic bath.<br><br>Energy transfer depends on bath temperature and power control.<br><br>Difficult to scale up for large-scale production.<br><br>Extended exposure to ultrasound can cause chemical changes in sensitive compounds.<br><br>Requires specialized equipment and careful parameter optimization.<br><br>[2,3,30]

NR: Not reported; NP: Normal Phase; MPLC: Medium Pressure Liquid Chromatography; RP: reverse phase; TLC: thin layer chromatography.

Table B. Summary of analytical methods for identification and determination of medicarpin.

Method	Analyzed sample	Purpose	Equipment and conditions	References	Pros	Cons
Capillary electrophoresis (CE)	Isolated (-)-medicarpin from alfalfa tissues and (+)-medicarpin from pathogen-infected peanut leaves	Identification	CE: CZE1000R 30 kV. D: UV-Vis (detection at 210 nm). Electrical field: 187.5 V/cm. Fused-silica capillaries (50 µm ID, 375 µm OD). Effective capillary length: 50 cm and total length: 80 cm	32	Suitable for several compound classes separation and for thermally unstable compounds.<br>Selective and efficient separation of complex mixtures.<br>Uses low amount of sample and solvents.<br>Can be coupled to different detectors and MS for compounds identification/quantification.<br>33,34	Low sensitivity for poor concentration samples.<br>Usually needs a prior sample preparation because of CE matrix intolerance.<br>Biomolecules analysis are limited as they can adsorb in the capillary surface.<br>35
GC	Mixtures of medicarpin and maackiain isolated from infected seedlings	Identification and quantification	D: Hydrogen flame ionization. SP: 183 x 0.2 cm id pyrex column, packed with 3.5% SE-30 on 110-120 mesh gas chrom Q.	7	Allows compounds separation with high resolution and sensitivity.<br>Can be coupled to different detectors and MS for compounds identification/quantification<br>Suitable for non-chromophore compounds.<br>Minimizes organic solvents use.<br>33	Limited to volatile and thermally stable compounds.<br>Non-volatile compounds should be derivatized to become volatile.<br>2
GC-MS	Isolated compounds from foliage	Identification	SP: 15 m DB-1 column. A 6-cm (i.d.) x 62-cm glass column, slurry packed with 45-500 g 60-200 mesh Silica gel. MS at 70 eV in the electron impact mode.	10	Provides high sensitivity and specificity.	Restricted to volatile or derivatized compounds.

	Extract with fungus infected chickpea seed	Identification	Finnigan 4015-GS-MC. SP: Fused-silica/quartz-capillary column (25 m x 0.32 mm) with a layer of SE-30-CB (0.27 um). Electron energy: 30 or 70 eV, emission 0.25 mA, source temperature: 250 °C, helium flow: 2 mL/min.	37	Ensures excellent separation of volatile and semi-volatile compounds.<br>Delivers structural information through fragmentation patterns.<br>Allows rapid identification using spectral libraries.<br>Supports both qualitative and quantitative analysis.<br>Offers wide applicability across scientific fields.<br>Provides reproducible and standardized results.<br>33,36	Requires complex and time-consuming sample preparation.<br>Involves high equipment and maintenance costs.<br>Destroys samples during analysis.<br>Susceptible to matrix interferences.<br>Ionization efficiency varies among compounds.<br>Demands specialized training for operation and interpretation.<br>2
GLC-MS	Trimethylsilyl (TMS)-medicarpin	Identification	Varian Aerograph Model 1400 GC. SP: Glass column ((1.5 m x 2 mm) packed with 1.5% OV-101 on 80-100 mesh Gas-Chrom Q coupled to a Micromass 70-70 mass spectrometer	25	See GC-MS and LC-MS sections	See GC-MS and LC-MS sections
HPLC	Extracts of seedlings or infected tissues	Identification	D: UV. SP: NP - 9.4 mm x 50 cm. Monitored at 280 nm.	8	Can be coupled to PDA detector, MS or NMR for samples identification/quantification.<br>A versatile and simple operating system.<br>Separation and detection of several analytes in a sample.<br>Suitable for separation of several compounds' classes.<br>Preparative HPLC is typically used for isolation or purification of large amounts of natural products.	Use of organic solvents as mobile phase which should be properly discarded.<br>For HPLC coupled to UV-vis detectors, the analyzed compounds should be chromophores, and for some compounds, the technique has low sensitivity.<br>Preparative HPLC demands high volume of solvents for mobile phase.<br>2,33
	Protoplasm extracts	Quantification	NR	21		
	Isolated compounds from foliage	Identification	D: UV	10		
	Fractions from cell suspension cultures	Identification and quantification by external standardization	D: DAD. SP: RP-18 - 250 x 4mm, 7 µm. MP: water (0.6% phosphoric acid) and acetonitrile in a gradient elution mode.	16		

2,33,38

Fungi agar plates	Identification	D: DAD. SP: C-18 analytical column. Monitored at 287 nm.	39
Seedlings and cell suspension	Identification	D: DAD. SP: RP-18 - 5.0 mm, 250, 4 mm column. MP: water (0.1% acetic acid) and acetonitrile in a gradient elution mode. Monitoring at 283 nm.	12
Methanolic extract from roots	Identification and quantification	D: DAD. SP: RP-18 column - 250 × 4 mm column; 5.0 mm. MP: water (0.1% acetic acid) and acetonitrile in a gradient elution mode. Detection at 283 nm. Quantification based on a calibration curve.	13
Synthetic Medicarpin	Identification	NR	40
Synthetized medicarpin	Identification	SP: Chiral column – 3 µm. MP: hexane and ethyl acetate (4:1). Run at 25℃.	41
Isolated medicarpin from plants	Identification	D: UV. SP: C18 column - 250 × 4.60 mm, 5 µm. MP: water (0.1% formic acid, v/v) and acetonitrile, in a gradient elution mode. Analysis at 280 nm. Run at 35℃.	22
Synthetic Medicarpin	Identification	NR	42
Extract with fungus infected chickpea seed	Identification/ quantification	NR	37
Isolated medicarpin or isomedicarpin from plant extract	Identification	SP: Whatman ODS-3 C18 column (4.6 x 250 mm). MP: Gradient elution with acetonitrile and water. Flow rate: 1 mL/min.	27
Extracts of leaflets, infected leaflets or	Identification/ Quantification	SP: Spherisorb ODS 10 um column (25 x 0.46 cm).	9

	treated with Copper (II) sulfate					
	Synthesized medicarpin	Identification	SP: X BridgeTM C18 column. MP: Acetonitrile:water (80:20). Flow rate: 0.35 mL/min. UV detection: 220 nm, 284 nm.	43		
	Isolated medicarpin enantiomers from infected leaves	Identification	SP: µPorasil column (10 um silica: 4.6 mm i.d. x 250 mm). MP: 1,2-dichloroethane-isso-PrOH (0.13%, isso-PrOH) on isocratic elution. Flow rate: 2.7 mL/min.	26		
	Enriched extract of a plant	Identification/ Quantification	SP: C18-ODS3 column (250 x 4.6 mm). MP: A gradient elution with acetonitrile and water (0.01% phosphoric acid). Flow rate: 1 mL/min. Injection volume: 20 uL.	17		
	Biosynthesized (-)-medicarpin/Isolated (-)-medicarpin from roots	Identification	SP: C18 column (250 x 4.6 mm, 5 um). MP: Gradient elution with acetonitrile and water (phosphoric acid 1%). Flow rate: 1.5 mL/min.	44		
HRMS	Synthesized medicarpin	Identification	ESI	43	Provides ultra-high mass accuracy for precise molecular formula determination.<br>See MS sections	See MS sections
	Isolated medicarpin from heartwood	Identification	ESI	28		
	Synthetized medicarpin	Identification	ESI. LTQ-FT-ICR-MS-7T spectrometer.	41		
	Isolated compounds from roots	Identification	ESI	14		

IR	Isolate medicarpin from plant sources	Identification	Performed on 10-25 mM in chloroform using 0.5 mm light path salt cells.	45	Simple and relatively quick method.<br>Provides information about functional groups present in the compound.<br>Requires minimal sample preparation.<br>Can be applied to solids, liquids, and some oils.<br>Useful for comparing known standards with unknown samples.<br>Cost-effective compared with more advanced techniques.<br>46	Limited sensitivity for compounds in low concentrations.<br>Overlapping absorption bands can make interpretation difficult.<br>Provides mainly functional group information, not full structural details.<br>Difficult to analyze complex mixtures without prior separation.<br>Requires experience for correct spectrum interpretation.<br>Interference from moisture, CO_2, or impurities can affect results.<br>46,47
	Medicarpin from medicarpin-β-D-glucoside isolated from *Medicago sativa* L.	Identification	JASCO IR-S	1		
LC-MS or LC-MS/MS	Isolated (+)-medicarpin from Jamaican multifloral propolis	Identification	ESI TOF. SP: reversed-phase analytical column (4.6 x 250 mm2, 5 µm)	19	Broad applicability to polar, non-volatile, and thermolabile compounds.<br>Requires little or no derivatization prior to analysis.<br>High sensitivity and selectivity for complex samples.<br>Enables simultaneous detection of a wide range of analytes.<br>LC–MS/MS offers superior structural information through tandem fragmentation.<br>LC–MS/MS provides enhanced selectivity, reducing matrix interferences.	Involves high instrument cost and complex maintenance.<br>Requires skilled operators for method development and data interpretation.<br>Ion suppression and matrix effects can compromise accuracy.<br>Limited robustness of spectral libraries compared to GC–MS.<br>LC–MS/MS may generate large, complex datasets that require advanced bioinformatics.<br>Method optimization can be time-consuming.<br>In-source fragmentation may complicate compound identification.
	Methanolic extract of plant tissues	Identification	ESI. MP: water (0.5% formic acid) and acetonitrile in a gradient elution mode. MicroTOF-Q mass spectrometer.	50		
	Plasma of rats treated with medicarpin	Quantification	Quantification using as internal standard 7-hydroxy isoflavone.	51		
	Methanolic extracts of leaf tissues	Identification	LC-ESI-MS/MS using a QTRAP 6500+ mass spectrometer	18		
	Synthesized medicarpin/medicarpin extracted from rat	Quantification	SP: X-bridge RP18 column (4.6 mm x 50 mm, 5.0 µm). MP: Isocratic mode with methanol and 10 mM ammonium acetate (pH 4.0) (80:20). Flow rate: 0.8 mL/min.	52		

	plasma, urine, feces and tissue matrices		Ionization source: ESI. MS: API 4000 QTRAP in MRM mode.		Suitable for both qualitative profiling and quantitative measurements.<br>Widely applied in metabolomics, proteomics, pharmacokinetics, and clinical studies.<br>48,49	48,49
MS	Isolate medicarpin from plant sources	Identification	ESI. Electron impact mass spectrometry (70eV).	45	Can be used for fast fingerprint analysis with direct insertion of samples.<br>Can be coupled to a prior chromatography technique, as HPLC and GC.<br>Several types of MS and some equipments can fragment the molecules (MS/MS). Can be used with different ionization sources.<br>Provides compounds structure information and it is used for their elucidation/identification.<br>One of the most sensitive and selective technique.<br>2,33,36	High cost of the equipment.<br>Can occur "Ion suppression" phenomenon caused by matrix components and signal reduction of the target.<br>53
	Isolated medicarpin from seedlings and cell suspension	Identification	ESI	12		
	Medicarpin from medicarpin-β-D-glucoside isolated from *Medicago sativa* L.	Identification	Hitachi RMU-6L) with ionization energy of 70 eV	1		
	Isolated medicarpin or isomedicarpin from plant extract	Identification	NR	27		
	Isolated medicarpin from infected pod endocarp tissue	Identification	NR	23		
	Extracts of leaflets, infected leaflets or treated with Copper (II) sulfate	Identification	NR	9		
	Enriched fractions of medicarpin from infected seedlings	Identification	NR	24		

	Isolated medicarpin enatiomers from infected leaves	Identification	NR	26		
	Synthesized medicarpin	Identification	Varian MAT 44 S coupled with a Varian Data 188	54		
	Biosynthesized (-)-medicarpin/Isolated (-)-medicarpin from roots	Identification	ZAB2-SE. Electron impact ionization mass espectra at 70 eV and acceleration voltage of 8 kV.	44		
	Isolated medicarpin from seedlings and cell suspension	Identification	APCI	12		
NMR	Isolate medicarpin from plant sources	Identification	300 MHz. In deuterated chloroform.	45	Non-destructive technique.<br>Can determine the number and types of hydrogen and carbon atoms.<br>Useful for identifying stereochemistry and isomerism.<br>Minimal sample preparation required.<br>Highly reproducible and reliable.<br>Can be used to analyze mixtures, sometimes without prior separation.<br>2,47	Requires relatively large amounts of pure sample compared with some other techniques.<br>Expensive equipment and maintenance costs.<br>Time-consuming for complex molecules.<br>Lower sensitivity for trace-level compounds.<br>Requires specialized training to interpret spectra.<br>Not ideal for very complex mixtures without separation techniques.<br>2,47
	Biosynthetically derived medicarpin	Identification	200 MHz. In deuterated chloroform.	55		
	Isolated compounds from foliage	Identification	Proton at 300 MHz. Carbon at 75.47 MHz. In deuterated chloroform.	10		
	Isolated substances from cell suspension cultures	Identification	300 MHz. In deuterated dimethyl sulfoxide.	16		
	Isolated medicarpin from seedlings and cell suspension	Identification	Proton at 400 MHz. Carbon at 50 MHz. In deuterated methanol.	12		
	Isolated medicarpin from heartwood	Identification	Proton at 400 MHz. Carbon at 100 MHz.	56		
	Synthetized medicarpin	Identification	Proton at 400 MHz. Carbon at 101 MHz.	41		
	Isolated medicarpin from plants	Identification	NR	22		

	Isolated compounds from roots	Identification	Proton at 600 MHz. Carbon at 150 MHz. In deuterated methanol.	14		
	Isolated substances from plant pods	Identification	Proton at 500 MHz or 700 MHz. Carbon at 125 MHz or 175 MHz.	15		
	Medicarpin from medicarpin-*β*-D-glucoside isolated from *Medicago sativa* L.	Identification	Proton at 100 MHz.	1		
	Isolated medicarpin or isomedicarpin from plant extract	Identification	Proton	27		
	Isolated medicarpin from infected pod endocarp tissue	Identification	Proton. In deuterated chloroform.	23		
	Extracts of leaflets, infected leaflets or treated with Copper (II) sulfate	Identification	Proton at 360 MHz. In deuterated acetone	9		
	Synthesized medicarpin	Identification	Proton at 400 MHz. Carbon at 100 MHz. In deuterated DMSO.	43		
	Isolated (+)-medicarpin from Jamaican multifloral propolis	Identification	Proton at 400 MHz. Carbon at 100 MHz. In deuterated chloroform	19		
Optical activity	Isolated substances from heartwood	Determination of the absolute configuration and conformation	Optical rotation	56	Provide information about chirality and absolute configuration of molecules.<br><br>Non-destructive techniques.	Limited to chiral compounds.<br><br>Provide only indirect structural information; cannot fully determine molecular structure.

	Isolated medicarpin from plants	Identification	Optical rotation	22	Require relatively small sample amounts.<br><br>Fast measurement with minimal sample preparation.<br><br>Useful for distinguishing enantiomers and diastereomers.<br><br>Can monitor conformational changes and interactions in solution (especially CD).<br><br>Complementary to other structural analysis techniques.<br><br>[57,58]	Sensitivity can be low for dilute samples.<br><br>Interpretation may be complex and requires expertise, especially for CD spectra.<br><br>Cannot provide quantitative information about overall chemical composition without standards.<br><br>Equipment can be expensive and less commonly available in some labs.<br><br>In CD, results can be affected by solvent, temperature, and concentration.<br><br>[57,58]
	Isolated compounds from roots	Determination of the absolute configuration	Optical rotation	14		
	Isolated substances from plant pods	Determination of the absolute configuration	Optical rotation on a Jasco P-1000 polarimeter	15		
	Isolated substances from plant pods	Determination of the absolute configuration and conformation	Circular dichroism on a J-815 spectrometer.	15		
	Medicarpin from medicarpin-β-D-glucoside isolated from plant	Identification	Optical rotation on a JASCO DIP-S polarimeter.	1		
	Isolated medicarpin from a infected pod endocarp tissue	Identification	Optical rotation	23		
	Extracts of leaflets, infected leaflets or treated with Copper (II) sulfate	Identification	Optical rotation	9		
	isolated (+)-medicarpin from Jamaican multifloral propolis	Identification	Optical rotation on a Jasco DIP 370 polarimeter	19		
	Isolated medicarpin enantiomers from infected leaves	Identification	Optical rotation	26		

	Biosynthesized (-)-medicarpin/Isolated (-)-medicarpin from roots	Identification	Circular dichroism	44		
TLC	Diffusates, tissue samples, culture filtrates and uninoculated leaves	Identification	SP: Silica gel plates. MP: three systems: *n*-pentane - ethyl ether - 80% acetic acid (75:25:1, v/v); chloroform with 2% (v/v) ethanol-methanol (100:4, v/v); propanol - ethyl acetate - water (7: 1 :2, v/v). R: *p*-nitroaniline.	5	Low-cost and fast analysis with simple sample preparation.<br>Used for quantitative and qualitative analysis of complex mixtures.<br>Fast comparison of samples with standards.<br>Can be coupled to MS and NMR to increase compounds identification.<br>TLC plates can be used for biological activities assays and extraction of the active compounds.<br>[38,59,60]	Flow rate is not controlled.<br>Compounds should fluoresce or absorb under UV light (360 and 254 nm, respectively) or should react with spraying reagents to become visible.<br>Samples with high content of substances are difficult to be characterized by TLC because the compounds could not separate appropriately<br>[2,36,38]
	Enriched fractions of medicarpin from seedlings	Identification	SP: Silica gel plates. MP: chloroform:carbon tetrachloride (3:1). R: UV fluorescence and *p*-nitroaniline.	6		
	Dichloromethane fraction of fungus infected seedlings	Identification	SP: Silica gel plates with fluorescent indicator.<br>MP: three systems pentane:ethyl ether:ethanol (75:25:5 v/v); toluene: ethyl acetate (8:1); chloroform:methanol (10:1).	7		
	Isolate medicarpin from plant sources	Identification	SP: Silica gel plates. MP: three systems toluene-ethyl acetate (1:1); chloroform-methanol (10:1); chloroform-methanol (25:1). R: *p*-nitroaniline and Gibbs reagent blue.	45		
	Extract with fungus infected seed	Identification	SP: Silica gel plates. MP: benzene:ethylacetate:petrolether: methanol = 6:4:3:2. R: diazotized *p*-nitroaniline.	37		
	Extracts of leaflets, infected leaflets or treated with Copper (II) sulfate	Identification	SP: Silica gel plates. MP: chloroform: MeOH (50:1); *n*-pentane: diethyl ether: glacial acetic acid (75:25:1, x2); benzene:methanol (9:1)	9		

	NR	Identification	SP: silica gel. MP: 19 different solvents-systems. R: Diazotized 4-nitro-aniline	61		
UHPLC	Fractions and isolated compounds from roots	Identification	D: DAD. SP: C18 column - 250 x 10 mm.	14	Provide superior chromatographic resolution and peak capacity.<br>Enable faster analysis with shorter run times compared to conventional HPLC.<br>Require smaller sample and solvent volumes, improving efficiency and sustainability.<br>Offer high sensitivity and selectivity when coupled with mass spectrometry.<br>Facilitate analysis of complex biological and environmental samples.<br>Allow reliable quantitation across a wide dynamic range.<br>62,63	Involve high instrument acquisition and maintenance costs.<br>Require columns with sub-2 µm particles, which are more expensive and less durable.<br>System operation demands highly trained personnel.<br>Susceptible to clogging due to small particle size and high backpressure.<br>Generate large datasets that require specialized software for interpretation.<br>Method transfer from conventional HPLC may be challenging.<br>Not always ideal for very large biomolecules due to pressure limitations.<br>62,63
UPLC -MS and -PDA	Isolated substances from plant pods	Identification	SP: C18 column. UPLC-QTOF-Ms with ESI source.	15		
UPLC-MS/MS	Biosynthetized medicarpin in enzymatic reactions and in *Saccharomyces cerevisiae* strain	Identification/ Quantification	UPLC-ESI-Q-TOF-MS/M. SP: C18 column - 100 x 2.1 mm, 1.7 µm). MP: water (0.1% formic acid) and acetonitrile in a gradient elution mode	31		
UV	Diffusates, tissue samples, culture filtrates and uninoculated leaves	Quantification	Samples were analyzed through a UV range of 220 - 350 nm on an UV spectrometer. The amount was determined using published extinction coefficients.	5	Simple and fast technique.<br>Results are simple to analyze and interpret.<br>Samples can often be recovered and reused.<br>Requires minimal sample preparation.<br>Cost-effective and widely available in most laboratories.	Limited to compounds containing chromophores; cannot detect non-absorbing molecules.<br>Limited structural information; cannot fully characterize compounds.<br>Low specificity in complex mixtures due to overlapping absorption bands.<br>Less sensitive compared with other modern techniques.
	Enriched fractions of medicarpin from seedlings	Quantification	The amount was determined using published extinction coefficients at 287 nm.	6		
	Extract with fungus infected seeds	Identification	NR	37		
	Medicarpin from medicarpin-β-D-	Identification	Cary Spectrometer Model 14	1		

	glucoside isolated from plant				Complementary to other structural analysis techniques. [47,64]	Requires pure or semi-pure samples for accurate quantitative analysis.<br>Interference from solvents or other chromophores may affect results.<br>Cannot provide stereochemical or detailed functional group information. [47,64]
	Isolated medicarpin from infected pod endocarp tissue	Identification	NR	23		
	Extracts of leaflets, infected leaflets or treated with Copper (II) sulfate	Identification	NR	9		
	Enriched fractions of medicarpin from infected seedlings	Identification/ Quantification	Detector: UV (250 - 320 nm)	24		
	Isolated medicarpin enantiomers from infected leaves	Identification	NR	26		
	NR	Quantification	Leitz-Unicam SP 8000 spectrophotometer at 287 nm	61		
	Synthesized medicarpin	Quantification	Zeiss DM 4 model at 285 nm	54		
	Biosynthesized (-)-medicarpin/Isolated (-)-medicarpin from roots	Identification	NR	44		

NR: Not reported; SP: stationary phase; MP: mobile phase; R: revealer; D: detector; RP: reverse phase; NP: normal phase.

(1) Sakagami, Y.; Kumai, S.; Suzuki, A. Isolation and Structure of Medicarpin-/s-d-Glucoside in Alfalfa. *Agric. Biol. Chem.* **1974**, *38* (5), 1031–1034. https://doi.org/10.1080/00021369.1974.10861260.

(2) Ponphaiboon, J.; Krongrawa, W.; Aung, W. W.; Chinatangkul, N.; Limmatvapirat, S.; Limmatvapirat, C. Electrospun Fiber Fabrication , and the Integration of Experimental Design : A Comprehensive Review. *Molecules* **2023**, *28* (13), 5163. https://doi.org/10.3390/molecules28135163.

(3) López-Olmos, C.; García-Valverde, M. T.; Hidalgo, J.; Ferrerio-Vera, C.; Medina, V. S. de. Comprehensive Comparison of Industrial Cannabinoid Extraction Techniques: Evaluation of the Most Relevant Patents and Studies at Pilot Scale. *Front. Nat. Prod.* **2022**, *1* (November), 1–39. https://doi.org/10.3389/fntpr.2022.1043147.

(4) Online, V. A.; Bucar, F.; Wube, A.; Schmid, M. Natural Product Isolation – How to Get from Biological. *Nat. Prod. Rep.* **2013**, *30*, 525–545. https://doi.org/10.1039/C3NP20106F.

(5) Duczek, L. J.; Higgins, V. J. The Role of Medicarpin and Maackiain in the Response of Red Clover Leaves to Helminthosporium Carbonum, Stemphylium Botryosum , and S. Sarcinaeforme. *Can. J. Bot.* **1976**, *54* (23), 2609–2619. https://doi.org/10.1139/b76-281.

(6) Khan, F. Z.; Milton, J. M. Some Factors Affecting the Production of Medicarpin and Sativan by Lucerne Leaflets in Response to Verticillium Albo-Atrum. *Physiol. Plant Pathol.* **1979**, *14* (1), 11–17. https://doi.org/10.1016/0048-4059(79)90019-5.

(7) Denny, T. P.; VanEtten, H. D. Tolerance by Nectria Haematococca MP VI of the Chickpea (Cicer Arietinum) Phytoalexins Medicarpin and Maackiain. *Physiol. Plant Pathol.* **1981**, *19* (3), 419–437. https://doi.org/10.1016/s0048-4059(81)80073-2.

(8) Vaziri, A. Stimulation of Oospore Production in Phytophthora Megasperma f. Sp. Medicaginis by Medicarpin. *Phytopathology* **1983**, *73* (5), 730. https://doi.org/10.1094/Phyto-73-730.

(9) Strange, R. N.; Edwards, C.; Ingham, J. L.; Cole, D. L.; Cavill, M. E.; Cooksey, C. J.; Garratt, P. J. Isolation of the Phytoalexin Medicarpin from Leaflets of Arachis Hypogaea and Related Species of the Tribe Aeschynomeneae. *Zeitschrift fur Naturforsch. - Sect. C J. Biosci.* **1985**, *40* (5–6), 313–316. https://doi.org/10.1515/znc-1985-5-605.

(10) Dornbos, D. L.; Spencer, G. F.; Miller, R. W. Medicarpin Delays Alfalfa Seed Germination and Seedling Growth The Objective of This Study Was to Identify a Compound (s) That May Be Responsible , at Least in Part , for Alfalfa Autotoxicity . We Found That Medicarpin Is a Compound Produced by Alfalfa . **1990**, *166*, 162–166.

(11) Guo, L.; Dixon, R. A.; Paiva, N. L. The ‘pterocarpan Synthase” of Alfalfa: Association and Co-induction of Vestitone Reductase and 7,2′-dihydroxy-4′-methoxy-isoflavanol (DMI) Dehydratase, the Two Final Enzymes in Medicarpin Biosynthesis. *FEBS Lett.* **1994**, *356* (2–3), 221–225. https://doi.org/10.1016/0014-5793(94)01267-9.

(12) Tsiri, D.; Chinou, I.; Halabalaki, M.; Haralampidis, K.; Ganis-Spyropoulos, C. The Origin of Copper-Induced Medicarpin Accumulation and Its Secretion from Roots of Young Fenugreek Seedlings Are Regulated by Copper Concentration. *Plant Sci.* **2009**, *176* (3), 367–374. https://doi.org/10.1016/j.plantsci.2008.12.001.

(13) Matsouka, I.; Beri, D.; Chinou, I.; Haralampidis, K.; Spyropoulos, C. G. Metals and Selenium Induce Medicarpin Accumulation and Excretion from the Roots of Fenugreek Seedlings: A Potential Detoxification Mechanism. *Plant Soil* **2011**, *343* (1–2), 235–245.

https://doi.org/10.1007/s11104-010-0714-6.

(14) Chern, C. M.; Lu, C. K.; Liou, K. T.; Wang, Y. H.; Tsai, K. C.; Chang, C. L.; Chang, C. C.; Shen, Y. C. Medicarpin Isolated from Radix Hedysari Ameliorates Brain Injury in a Murine Model of Cerebral Ischemia. *J. Food Drug Anal.* **2021**, *29* (4), 581–605. https://doi.org/10.38212/2224-6614.3377.

(15) Oh, J. M.; Jang, H. J.; Kang, M. G.; Mun, S. K.; Park, D.; Hong, S. J.; Kim, M. H.; Kim, S. Y.; Yee, S. T.; Kim, H. Medicarpin and Homopterocarpin Isolated from Canavalia Lineata as Potent and Competitive Reversible Inhibitors of Human Monoamine Oxidase-B. *Molecules* **2023**, *28* (1). https://doi.org/10.3390/molecules28010258.

(16) Weidemann, C.; Tenhaken, R.; Höhl, U.; Barz, W. Medicarpin and Maackiain 3-O-Glucoside-6?-O-Malonate Conjugates Are Constitutive Compounds in Chickpea (Cicer Arietinum L.) Cell Cultures. *Plant Cell Rep.* **1991**, *10* (6–7), 762–765. https://doi.org/10.1007/BF00193162.

(17) Ghelich, S.; Zarinkamar, F.; Mohammad Soltani, B.; Niknam, V. Effect of Lead Treatment on Medicarpin Accumulation and on the Gene Expression of Key Enzymes Involved in Medicarpin Biosynthesis in Medicago Sativa L. *Environ. Sci. Pollut. Res.* **2014**, *21* (24), 14091–14098. https://doi.org/10.1007/s11356-014-3335-4.

(18) Gupta, A.; Awasthi, P.; Sharma, N.; Parveen, S.; Vats, R. P.; Singh, N.; Kumar, Y.; Goel, A.; Chandran, D. Medicarpin Confers Powdery Mildew Resistance in Medicago Truncatula and Activates the Salicylic Acid Signalling Pathway. *Mol. Plant Pathol.* **2022**, *23* (7), 966–983. https://doi.org/10.1111/mpp.13202.

(19) Williams, D.; Perry, D.; Carraway, J.; Simpson, S.; Uwamariya, P.; Christian, O. E. Antigonococcal Activity of (+)-Medicarpin. *ACS Omega* **2021**, *6* (23), 15274–15278. https://doi.org/10.1021/acsomega.1c01590.

(20) Lucy, M. C.; Matthews, P. S.; VanEtten, H. D. Metabolic Detoxification of the Phytoalexins Maackiain and Medicarpin by Nectria Haematococca Field Isolates: Relationship to Virulence on Chickpea. *Physiol. Mol. Plant Pathol.* **1988**, *33* (2), 187–199. https://doi.org/10.1016/0885-5765(88)90019-7.

(21) Kessmann, H.; Choudhary, A.; Dixon, R. Stress Responses in Alfalfa (Medicago Sativa L.) III. Induction of Medicarpin and Cytochrome P450 Enzyme Activities in Elicitor-Treated Cell Suspension Cultures and Protoplasts. *Plant Cell Rep.* **1990**, *9* (1), 47–50. https://doi.org/10.1007/BF00232132.

(22) Wang, H. Y.; Li, T.; Ji, R.; Xu, F.; Liu, G. X.; Li, Y. L.; Shang, M. Y.; Cai, S. Q. Metabolites of Medicarpin and Their Distributions in Rats. *Molecules* **2019**, *24* (10). https://doi.org/10.3390/molecules24101966.

(23) Hargreaves, J. A.; Mansfield, J. W.; Coxon, D. T. Identification of Medicarpin as a Phytoalexin in the Broad Bean Plant (Vicia Faba L.). *Nature* **1976**, *262* (5566), 318–319. https://doi.org/10.1038/262318a0.

(24) Vaziri, A.; Keen, N. T.; Erwin, D. C. Correlation of Medicarpin Production with Resistance to Phytophthora Megasperma f. Sp. Medicaginis in Alfalfa Seedlings. *The American Phytopathological Society*. 1981, pp 1235–1238.

(25) Woodward, M. D. Identification of the Biosynthetic Precursors of Medicarpin in Inoculation Droplets on White Clover. *Physiol. Plant Pathol.* **1981**, *18* (1), 33–39. https://doi.org/10.1016/s0048-4059(81)80051-3.

(26) VanEtten, H. D.; Matthews, P. S.; Mercer, E. H. (+)-Maackiain and (+)-Medicarpin as Phytoalexins in Sophora Japonica and Identification of the (-) Isomers by Biotransformation. *Phytochemistry* **1983**, *22* (10), 2291–2295. https://doi.org/10.1016/S0031-9422(00)80164-8.

(27) Miller, D. K.; Sadowski, S.; Han, G. Q.; Joshua, H. Identification and Isolation of Medicarpin and a Substituted Benzofuran as Potent Leukotriene Inhibitors in an Anti-Inflammatory Chinese Herb. *Prostaglandins, Leukot. Essent. Fat. Acids* **1989**, *38* (2), 137–143. https://doi.org/10.1016/0952-3278(89)90098-7.

(28) Martínez-Sotres, C.; López-Albarrán, P.; Cruz-de-León, J.; García-Moreno, T.; Rutiaga-Quiñones, J. G.; Vázquez-Marrufo, G.; Tamariz-Mascarúa, J.; Herrera-Bucio, R. Medicarpin, an Antifungal Compound Identified in Hexane Extract of Dalbergia Congestiflora Pittier Heartwood. *Int. Biodeterior. Biodegradation* **2012**, *69*, 38–40. https://doi.org/10.1016/j.ibiod.2011.11.016.

(29) Quitério, E.; Grosso, C.; Ferraz, R.; Delerue-Matos, C.; Soares, C. A Critical Comparison of the Advanced Extraction Techniques Applied to Obtain Health-Promoting Compounds from Seaweeds. *Mar. Drugs* **2022**, *20* (11), 1–40. https://doi.org/10.3390/md20110677.

(30) Bitwell, C.; Sen, S.; Luke, C. A Review of Modern and Conventional Extraction Techniques and Their Applications for Extracting Phytochemicals from Plants. *Sci. African* **2023**, *19*, e01585. https://doi.org/10.1016/j.sciaf.2023.e01585.

(31) Lu, C.; Du, R.; Fu, H.; Zhang, J.; Zhao, M.; Wei, Y.; Lin, W. Heterologous Biosynthesis of Medicarpin Using Engineered Saccharomyces Cerevisiae. *Synth. Syst. Biotechnol.* **2023**, *8* (4), 749–756. https://doi.org/10.1016/j.synbio.2023.11.003.

(32) Allen, D. J.; Gray, J. C.; Paiva, N. L.; Smith, J. T. An Enantiomeric Assay for the Flavonoids Medicarpin and Vestitone Using Capillary Electrophoresis. *Electrophoresis* **2000**, *21* (10), 2051–2057. https://doi.org/10.1002/1522-2683(20000601)21:10<2051::AID-ELPS2051>3.0.CO;2-6.

(33) Chew, Y.-L.; Khor, M.-A.; Lim, Y.-Y. Choices of Chromatographic Methods as Stability Indicating Assays for Pharmaceutical Products: A Review. *Heliyon* **2021**, *7* (3), e06553. https://doi.org/10.1016/j.heliyon.2021.e06553.

(34) Sastre Toraño, J.; Ramautar, R.; de Jong, G. Advances in Capillary Electrophoresis for the Life Sciences. *J. Chromatogr. B* **2019**, *1118–1119*, 116–136. https://doi.org/10.1016/j.jchromb.2019.04.020.

(35) Ramos-Payán, M.; Ocaña-Gonzalez, J. A.; Fernández-Torres, R. M.; Llobera, A.; Bello-López, M. Á. Recent Trends in Capillary Electrophoresis for Complex Samples Analysis: A Review. *Electrophoresis* **2018**, *39* (1), 111–125. https://doi.org/10.1002/elps.201700269.

(36) Sawaya, A. C. H. F.; Barbosa da Silva Cunha, I.; Marcucci, M. C. Analytical Methods Applied to Diverse Types of Brazilian Propolis. *Chem. Cent. J.* **2011**, *5* (1), 1–10. https://doi.org/10.1186/1752-153X-5-27.

(37) Kraft, B.; Schwenen, L.; Stöckl, D.; Barz, W. Degradation of the Pterocarpan Phytoalexin Medicarpin by Ascochyta Rabiei. *Arch.*

Microbiol. **1987**, *147* (2), 201–206. https://doi.org/10.1007/BF00415285.

(38) Bucar, F.; Wube, A.; Schmid, M. Natural Product Isolation – How to Get from Biological Material to Pure Compounds. *Nat. Prod. Rep.* **2013**, *30* (4), 525. https://doi.org/10.1039/c3np20106f.

(39) Blount, J. W.; Dixon, R. A.; Paiva, N. L. Stress Responses in Alfalfa (Medicago Sativa L.) XVI. Antifungal Activity of Medicarpin and Its Biosynthetic Precursors; Implications for the Genetic Manipulation of Stress Metabolites. *Physiol. Mol. Plant Pathol.* **1992**, *41* (5), 333–349. https://doi.org/10.1016/0885-5765(92)90020-V.

(40) Dixit, M.; Raghuvanshi, A.; Gupta, C. P.; Kureel, J.; Mansoori, M. N.; Shukla, P.; John, A. A.; Singh, K.; Purohit, D.; Awasthi, P.; Singh, D.; Goel, A. Medicarpin, a Natural Pterocarpan, Heals Cortical Bone Defect by Activation of Notch and Wnt Canonical Signaling Pathways. *PLoS One* **2015**, *10* (12), e0144541. https://doi.org/10.1371/journal.pone.0144541.

(41) Yang, X.; Zhao, Y.; Hsieh, M. T.; Xin, G.; Wu, R. T.; Hsu, P. L.; Horng, L. Y.; Sung, H. C.; Cheng, C. H.; Lee, K. H. Total Synthesis of (+)-Medicarpin. *J. Nat. Prod.* **2017**, *80* (12), 3284–3288. https://doi.org/10.1021/acs.jnatprod.7b00741.

(42) Mansoori, M. N.; Raghuvanshi, A.; Shukla, P.; Awasthi, P.; Trivedi, R.; Goel, A.; Singh, D. Medicarpin Prevents Arthritis in Post-Menopausal Conditions by Arresting the Expansion of TH17 Cells and pro-Inflammatory Cytokines. *Int. Immunopharmacol.* **2020**, *82* (February), 106299. https://doi.org/10.1016/j.intimp.2020.106299.

(43) Sharma, K.; Awasthi, P.; Prakash, R.; Khanka, S.; Bajpai, R.; Sahasrabuddhe, A. A.; Goel, A.; Singh, D. Maintenance of Increased Bone Mass after PTH Withdrawal by Sequential Medicarpin Treatment via Augmentation of CAMP-PKA Pathway. *J. Cell. Biochem.* **2022**, *123* (11), 1762–1779. https://doi.org/10.1002/jcb.30313.

(44) Guo, L.; Dixon, R. A.; Paiva, N. L. Conversion of Vestitone to Medicarpin in Alfalfa (Medicago Sativa L.) Is Catalyzed by Two Independent Enzymes: Identification, Purification, and Characterization of Vestitone Reductase and 7,2′-Dihydroxy-4′-Methoxyisoflavanol Dehydratase. *J. Biol. Chem.* **1994**, *269* (35), 22372–22378. https://doi.org/10.1016/s0021-9258(17)31799-4.

(45) Denny, T. P.; VanEtten, H. D. Metabolism of the Phytoalexins Medicarpin and Maackiain by Fusarium Solani. *Phytochemistry* **1982**, *21* (5), 1023–1028. https://doi.org/10.1016/S0031-9422(00)82409-7.

(46) Cozzolino, D. Benefits and Limitations of Infrared Technologies in Omics Research and Development of Natural Drugs and Pharmaceutical Products. *Drug Dev. Res.* **2012**, *73* (8), 504–512. https://doi.org/10.1002/ddr.21043.

(47) Mondal, S.; Das, M.; Debnath, S.; Sarkar, B. K. An Overview of Extraction , Isolation and Characterization Techniques of Phytocompounds from Medicinal Plants. *Nat. Prod. Res.* **2024**, *0* (0), 1–23. https://doi.org/10.1080/14786419.2024.2426059.

(48) Nasiri, A.; Jahani, R.; Mokhtari, S.; Yazdanpanah, H.; Daraei, B.; Faizi, M.; Kobarfard, F. Overview, Consequences, and Strategies for Overcoming Matrix Effects in LC-MS Analysis: A Critical Review. *Analyst* **2021**, *146* (20), 6049–6063. https://doi.org/10.1039/d1an01047f.

(49) Chen, Y.; Yu, H.; Wu, H.; Pan, Y.; Wang, K.; Jin, Y.; Zhang, C. Characterization and Quantification by LC-MS/MS of the Chemical Components of the Heating Products of the Flavonoids Extract in Pollen Typhae for Transformation Rule Exploration. *Molecules* **2015**, *20* (10), 18352–18366. https://doi.org/10.3390/molecules201018352.

(50) Biała, W.; Banasiak, J.; Jarzyniak, K.; Pawela, A.; Jasiński, M. Medicago Truncatula ABCG10 Is a Transporter of 4-Coumarate and Liquiritigenin in the Medicarpin Biosynthetic Pathway. *J. Exp. Bot.* **2017**, *68* (12), 3231–3241. https://doi.org/10.1093/jxb/erx059.

(51) Bhargavan, B.; Singh, D.; Gautam, A. K.; Mishra, J. S.; Kumar, A.; Goel, A.; Dixit, M.; Pandey, R.; Manickavasagam, L.; Dwivedi, S. D.; Chakravarti, B.; Jain, G. K.; Ramachandran, R.; Maurya, R.; Trivedi, A.; Chattopadhyay, N.; Sanyal, S. Medicarpin, a Legume Phytoalexin, Stimulates Osteoblast Differentiation and Promotes Peak Bone Mass Achievement in Rats: Evidence for Estrogen Receptor β-Mediated Osteogenic Action of Medicarpin. *J. Nutr. Biochem.* **2012**, *23* (1), 27–38. https://doi.org/10.1016/j.jnutbio.2010.11.002.

(52) Taneja, I.; Raju, K. S. R.; Challagundla, M.; Raghuvanshi, A.; Goel, A.; Wahajuddin, M. LC-ESI-MS/MS Method for Bioanalytical Determination of Osteogenic Phytoalexin, Medicarpin, and Its Application to Preliminary Pharmacokinetic Studies in Rats. *J. Chromatogr. B Anal. Technol. Biomed. Life Sci.* **2015**, *1001*, 9–16. https://doi.org/10.1016/j.jchromb.2015.06.025.

(53) Wu, A. H. B.; French, D. Implementation of Liquid Chromatography/Mass Spectrometry into the Clinical Laboratory. *Clin. Chim. Acta* **2013**, *420*, 4–10. https://doi.org/10.1016/j.cca.2012.10.026.

(54) Weltring, K. M.; Barz, W.; Dewick, P. M. Degradation of 3,9-Dimethoxypterocarpan and Medicarpin by Fusarium Fungi. *Arch. Microbiol.* **1981**, *130* (5), 381–384. https://doi.org/10.1007/BF00414604.

(55) Banks, S. W.; Steele, M. J.; Ward, D.; Dewick, P. M. Stereochemistry of Isoflavone Reduction during the Biosynthesis of (+)-and (-)-Pterocarpans: 2H n.m.r. Studies on the Biosynthesis of (+)-Pisatin and (-)-Medicarpin. *J. Chem. Soc. Chem. Commun.* **1982**, No. 3, 157–158. https://doi.org/10.1039/C39820000157.

(56) Martínez-Sotres, C.; López-Albarrán, P.; Cruz-de-León, J.; García-Moreno, T.; Rutiaga-Quiñones, J. G.; Vázquez-Marrufo, G.; Tamariz-Mascarúa, J.; Herrera-Bucio, R. Medicarpin, an Antifungal Compound Identified in Hexane Extract of Dalbergia Congestiflora Pittier Heartwood. *Int. Biodeterior. Biodegrad.* **2012**, *69*, 38–40. https://doi.org/10.1016/j.ibiod.2011.11.016.

(57) Zhu, S.; Sun, M. Electronic Circular Dichroism and Raman Optical Activity : Principle and Applications Electronic Circular Dichroism and Raman Optical Activity : Principle and Applications. *Appl. Spectrosc. Rev.* **2020**, *0* (0), 1–35. https://doi.org/10.1080/05704928.2020.1831523.

(58) Pereda, R.; Elihu, M.; Lucero, B.; Fructuoso, M.; Fragoso, M. *From Relative to Absolute Stereochemistry of Secondary Metabolites : Applications in Plant Chemistry*; Springer International Publishing, 2023. https://doi.org/10.1007/s43450-022-00333-y.

(59) Mohammad, A.; Moheman, A. TLC/HPTLC in Biomedical Applications. In *High-Performance Thin-Layer Chromatography (HPTLC)*; Springer Berlin Heidelberg: Berlin, Heidelberg, 2011; pp 151–178. https://doi.org/10.1007/978-3-642-14025-9_10.

(60) Sawaya, A. C. H. F.; Barbosa da Silva Cunha, I.; Marcucci, M. C. Analytical Methods Applied to Diverse Types of Brazilian Propolis.

Chem. Cent. J. **2011**, *5* (1), 27. https://doi.org/10.1186/1752-153X-5-27.

(61) Weltring, K. M.; Barz, W. Degradation of 3, 9-Dimethoxypterocarpan and Medicarpin by Fusarium Proliferatum. *Zeitschrift fur Naturforsch. - Sect. C J. Biosci.* **1980**, *35* (5–6), 399–405. https://doi.org/10.1515/znc-1980-5-609.

(62) Nahar, L.; Onder, A.; Sarker, S. D. A Review on the Recent Advances in HPLC, UHPLC and UPLC Analyses of Naturally Occurring Cannabinoids (2010–2019). *Phytochem. Anal.* **2020**, *31* (4), 413–457. https://doi.org/10.1002/pca.2906.

(63) Nahar, L.; Sarker, S. D. UPLC in Phytochemical Analysis. *Trends Phytochem. Res.* **2019**, *3* (1), 1–2.

(64) Altemimi, A.; Lakhssassi, N.; Baharlouei, A.; Watson, D. G. And Identification of Bioactive Compounds from Plant Extracts. **2017**. https://doi.org/10.3390/plants6040042.
